# SOD2 is a regulator of proteasomal degradation promoting an adaptive cellular starvation response

**DOI:** 10.1016/j.celrep.2025.115434

**Published:** 2025-03-24

**Authors:** Nurul Khalida Ibrahim, Sabine Schreek, Buesra Cinar, Anna Sophie Stasche, Su Hyun Lee, Andre Zeug, Tim Dolgner, Julia Niessen, Evgeni Ponimaskin, Halyna Shcherbata, Beate Fehlhaber, Jean-Pierre Bourquin, Beat Bornhauser, Martin Stanulla, Andreas Pich, Alejandro Gutierrez, Laura Hinze

**Affiliations:** 1 Department of Pediatric Hematology and Oncology, Hannover Medical School, 30625 Hannover, Germany; 2 Division of Hematology/Oncology, Boston Children’s Hospital, Harvard Medical School, Boston, MA 02115, USA; 3 Department of Cellular Neurophysiology, Hannover Medical School, 30625 Hannover, Germany; 4 Department of Cell Biochemistry, Hannover Medical School, 30625 Hannover, Germany; 5 Mount Desert Island Biological Laboratory, Bar Harbor, ME 04609, USA; 6 Department of Pediatric Hematology/Oncology, University Children’s Hospital, 8032 Zurich, Switzerland; 7 Institute of Toxicology, Research Core Unit – Proteomics, Hannover Medical School, 30625 Hannover, Germany; 8 Department of Oncology, Dana-Farber Cancer Institute, Harvard Medical School, Boston, MA 02215, USA; 9 Department of Pediatric Oncology, St. Jude Children’s Research Hospital, Memphis, TN 38105, USA; 10 Lead contact

## Abstract

Adaptation to changes in amino acid availability is crucial for cellular homeostasis, which requires an intricate orchestration of involved pathways. Some cancer cells can maintain cellular fitness upon amino acid shortage, which has a poorly understood mechanistic basis. Leveraging a genome-wide CRISPR-Cas9 screen, we find that superoxide dismutase 2 (SOD2) has a previously unrecognized dismutase-independent function. We demonstrate that SOD2 regulates global proteasomal protein degradation and promotes cell survival under conditions of metabolic stress in malignant cells through the E3 ubiquitin ligases UBR1 and UBR2. Consequently, inhibition of SOD2-mediated protein degradation highly sensitizes different cancer entities, including patient-derived xenografts, to amino acid depletion, highlighting the pathophysiological relevance of our findings. Our study reveals that SOD2 is a regulator of proteasomal protein breakdown upon starvation, which serves as an independent catabolic source of amino acids, a mechanism co-opted by cancer cells to maintain cellular fitness.

## In brief

Ibrahim et al. show that SOD2 has a dismutase-independent function by regulating proteasomal degradation through UBR1 and UBR2. This SOD2-driven protein breakdown enhances cancer cell survival upon starvation by creating an alternative source of amino acids. In leukemia, inhibition of SOD2 sensitizes cells to asparaginase treatment, demonstrating its therapeutic potential.

## Graphical abstract

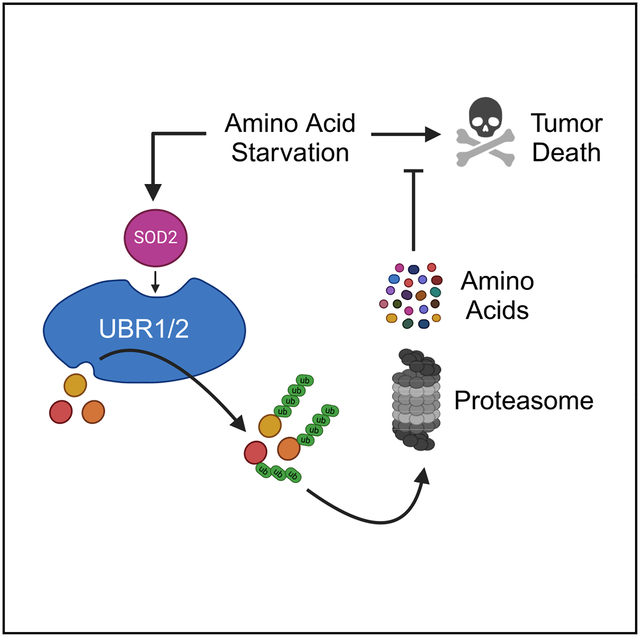

## INTRODUCTION

The survival capacity of cells depends on their ability to withstand or evade variable cellular stressors that dysregulate cellular homeostasis. Exploiting the dependency of cancer cells on amino acid availability offers therapeutic avenues, for instance, through the use of asparaginase, which depletes the non-essential amino acid asparagine. This potent activity of asparaginase in hematopoietic neoplasms has led to its integral role in the first-line standard of care for lymphoblastic leukemias.^1–4^ Dose intensification of asparaginase has led to improved outcomes in T cell and B cell acute lymphoblastic leukemias^1,5–7^; however, resistance to asparaginase-based therapies is associated with poor prognosis, and effective treatment options remain limited for many of these patients.^8^

The molecular mechanisms that allow mammalian cells to maintain cellular fitness upon asparagine starvation remain incompletely understood. Eukaryotic cells are well known to respond to amino acid starvation by inhibiting protein synthesis to reduce amino acid consumption,^9,10^ increase *de novo* amino acid biosynthesis and transport, and stimulate lysosomal protein degradation as a catabolic source of amino acids.^11–14^ However, the role of the ubiquitin-proteasome system in this context is still evolving. The proteasome is a highly sophisticated protease complex that can serve as a source of proteinogenic amino acids. However, the mechanisms that regulate proteasomal degradation under conditions of metabolic stress remain poorly understood.

Here, we show that the inhibition of the superoxide dismutase 2 (SOD2) sensitizes different cancer entities, including leukemia and colorectal cancer cells, to amino acid starvation independently of known SOD2-associated pathways, including reactive oxygen species (ROS) signaling. Instead, we demonstrate that SOD2 has a previously unrecognized dismutase-independent function and regulates protein degradation through the E3 ubiquitin ligases UBR1 and UBR2 upon starvation. While UBRs have been mainly associated with the Arg/N-degron pathway, which targets proteins for degradation by recognizing their N-terminal or internal degrons,^15–20^ we here show that SOD2 does not appear to mediate degradation of particular N-degron substrates, but instead regulates proteasomal protein degradation as a starvation response. Intriguingly, inhibition of SOD2 or UBRs highly sensitized drug-resistant patient-derived xenograft (PDX) leukemia specimens to asparaginase. Thus, SOD2-regulated protein degradation promotes cancer cell fitness upon amino acid depletion, reflecting an adaptive mechanism to maintain metabolic homeostasis.

We propose a model in which the interaction of SOD2 and UBRs can adaptively trigger protein breakdown as an independent catabolic source of amino acids, a mechanism co-opted by cancer cells to maintain cellular fitness upon starvation. This study highlights the unique role of these biomolecules within a distinct pathway relevant to cell fitness, protein metabolism, and cancer therapy.

## RESULTS

### SOD2 inhibition sensitizes leukemia cells to asparagine depletion

**Figure 1.**
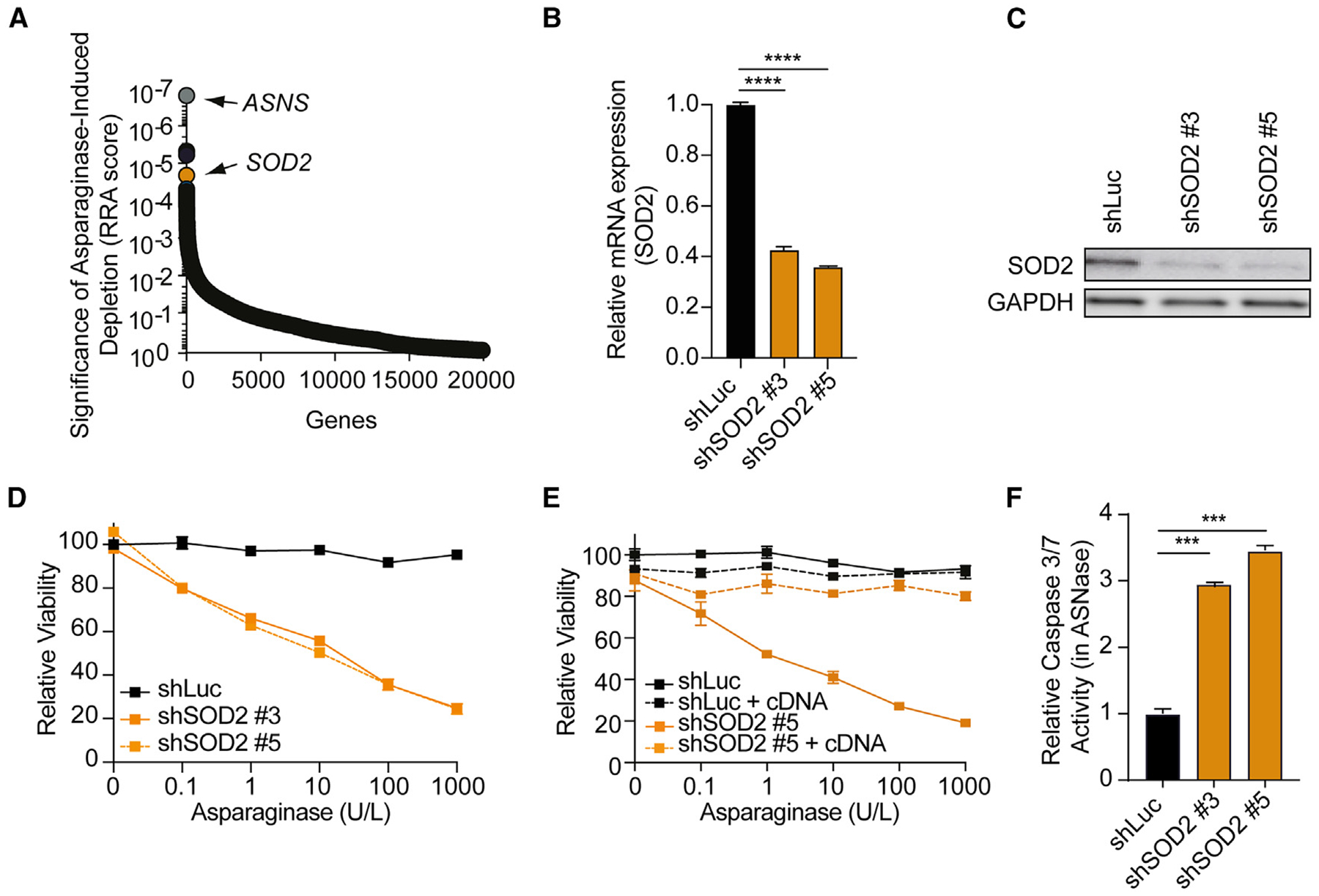
SOD2 inhibition sensitizes leukemia cells to asparagine depletion (A) Significance of *SOD2* depletion upon asparaginase treatment was assessed by robust ranking aggregation (RRA) score calculated using the MAGeCK analysis.^23^ Note that details on the CRISPR-Cas9 screen can be found in.^21^ (B) CCRF-CEM cells were transduced with indicated shRNAs, and knockdown efficiency was assessed with RT-qPCR analysis in biological duplicates. (C) CCRF-CEM cells were transduced with shLuc, shSOD2 #3, or shSOD2 #5. Protein levels of SOD2 and GAPDH were assessed by western blot analysis. (D) CCRF-CEM cells were transduced with shLuc, shSOD2 #3, or shSOD2 #5 and treated with indicated asparaginase doses in biological triplicates. Relative viability was assessed after 8 days of treatment. All cell counts were normalized to shLuc-transduced, vehicle-treated cells. (E) CCRF-CEM cells were transduced with indicated shRNAs. Cells expressing shRNAs in the presence or absence of SOD2 cDNA were subsequently treated with the indicated doses of asparaginase in biological triplicates. Relative viability was assessed after 8 days of treatment. Cell counts were normalized as in (D). (F) CCRF-CEM cells were transduced with indicated shRNAs and treated with 100 U/L asparaginase for 48 h, and caspase 3/7 activity was assessed in biological duplicates. All error bars represent SEM. ****p ≤ 0.0001, ***p ≤ 0.001 by one-way ANOVA Dunnett’s adjustment for multiple comparisons (B and F). See also Table S1 and Figure S1.

Leveraging a genome-wide CRISPR-Cas9 loss-of-function screen in the resistant T cell acute lymphoblastic leukemia (T-ALL) cell line CCRF-CEM^21^ to identify molecular pathways that drive response and resistance to asparaginase, we could previously show that WNT-induced inhibition of GSK3-dependent protein degradation (WNT/STOP) sensitizes resistant cancer cells to asparaginase.^21,22^ In this screen, we also found the gene *SOD2* among the top hits (Figure 1A), which attracted our interest for further investigation as it has not previously been linked to asparaginase response in resistant leukemias. Of note, analysis of guide RNA-level results revealed that all six guide RNAs targeting *SOD2* were significantly depleted in asparaginase-treated cells (Figure S1A; Table S1), making it unlikely to reflect a false-positive hit.

To validate that loss of *SOD2* sensitizes leukemia cells to asparaginase, we first induced an shorth hairpin RNA (shRNA)-mediated knockdown of SOD2 (shSOD2), which resulted in efficient gene silencing as assessed by RT-qPCR (Figure 1B) and western blot (Figure 1C). Inhibition of SOD2 by RNA interference induced a striking asparaginase sensitization (Figure 1D), which could be rescued by expression of the SOD2 cDNA (Figure 1E). Additionally, inhibition of SOD2 potentiated asparaginase-induced mitochondrial apoptosis (Figure 1F). Thus, inhibition of SOD2 mediates sensitization to asparaginase in these cells.

### SOD2 inhibition sensitizes different cancer entities to asparaginase-induced cytotoxicity

**Figure 2.**
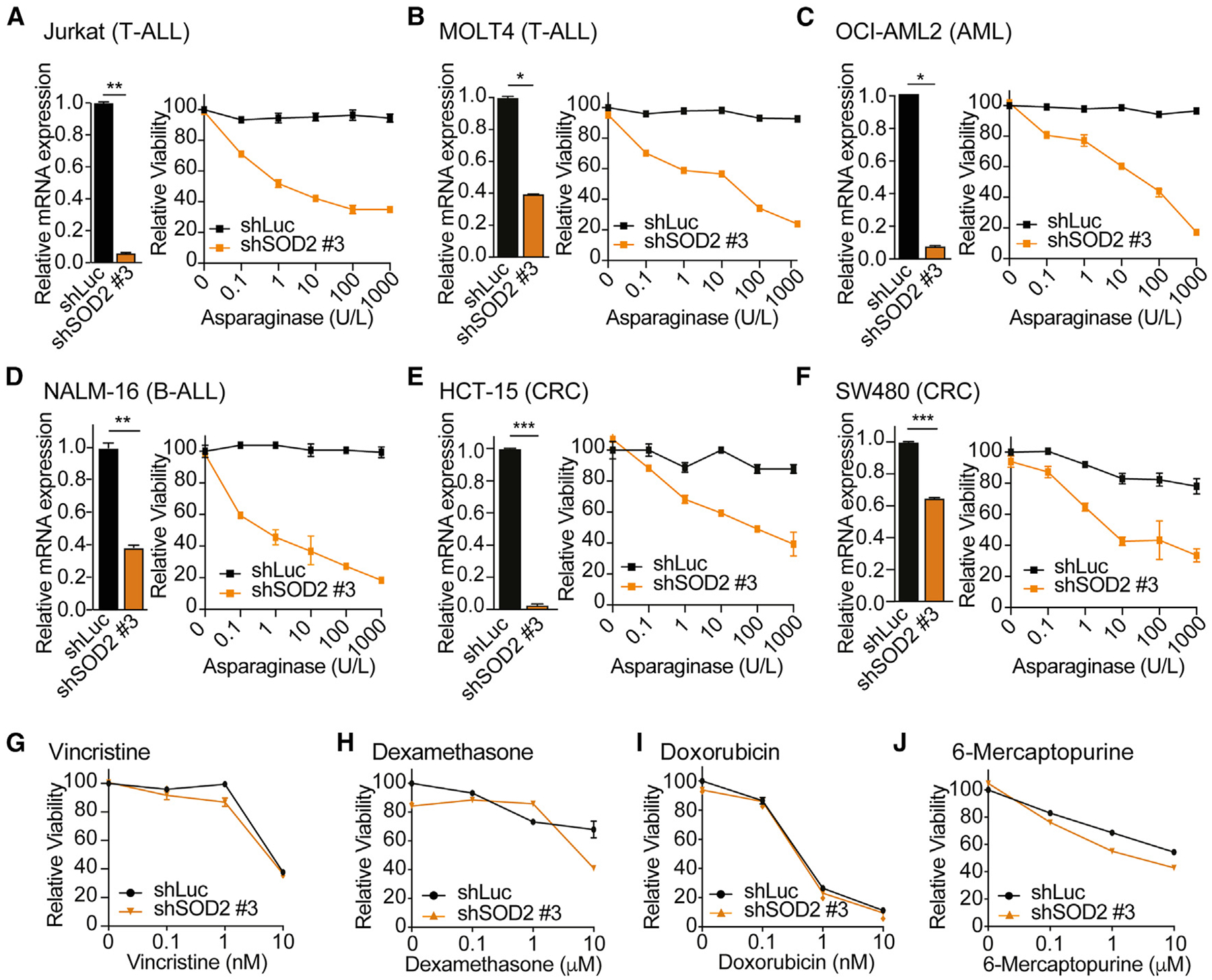
SOD2 inhibition sensitizes different cancer entities to asparaginase-induced cytotoxicity (A–F, left) Jurkat, MOLT4, OCI-AML2, NALM-16, HCT-15, and SW480 cells were transduced with the indicated shRNAs, and the knockdown efficiency of SOD2 was assessed by RT-qPCR analysis in biological duplicates. (A–F, right) Jurkat, MOLT4, OCI-AML2, NALM-16 HCT-15, and SW480 cells were treated with the indicated doses of asparaginase in biological triplicates. After 8 days of treatment, relative viability was assessed. Cell counts were normalized to shLuc-transduced, vehicle-treated cells. (G–J) Jurkat cells were transduced with the indicated shRNAs and treated with the indicated doses of vincristine, dexamethasone, doxorubicin, and 6-mercaptopurine in biological duplicates. After 8 days of treatment, relative viability was assessed. Cell counts were normalized as in (A–F). All error bars represent SEM. ***p ≤ 0.001, **p ≤ 0.01, *p < 0.05 by two-sided Student’s t test with Welch adjustment (A–F, left). See also Figure S1 and Table S2.

To exclude the possibility that the observed sensitization is a cell line-specific effect, we used a panel of cell lines from different cancer entities (Table S2). Knockdown of SOD2 induced a significant reduction in viability upon treatment with asparaginase (Figures 2A–2F) but did not sensitize leukemic cells to other commonly used leukemia chemotherapeutic agents (Figures 2G–2J).

SODs have been reported to be located in different cellular compartments: SOD1 in the cytosol and mitochondria, SOD2 mainly in the mitochondria, and SOD3 in the extracellular compartment (reviewed by Fukai et al.^24^). This prompted us to test whether the sensitization phenotype is selective to SOD2, or whether it could be phenocopied by the inhibition of other isoforms. We started by transducing shRNAs selectively targeting the SOD1 or SOD3 isoforms (Figures S1B–S1D). Knockdown of SOD1 (Figure S1E) exhibited high baseline toxicity in all transduced cell lines in the absence of asparaginase treatment (Figure S1F), in line with the general toxicity attributed to SOD1 inhibition from previous work.^25–27^ Similarly, the knockdown of the extracellularly located isoform SOD3 failed to induce asparaginase sensitization (Figure S1G).

### SOD2-regulated asparaginase response is independent of known SOD2-associated pathways

We then asked how SOD2 inhibition sensitizes cancer cells to asparagine depletion. The activity of SODs has been extensively related to oxidative stress (reviewed by Fukai et al.^24^ and Miao et al.^28^), thus raising the possibility that SOD2-mediated asparaginase sensitization is related to changes in ROS.

**Figure 3.**
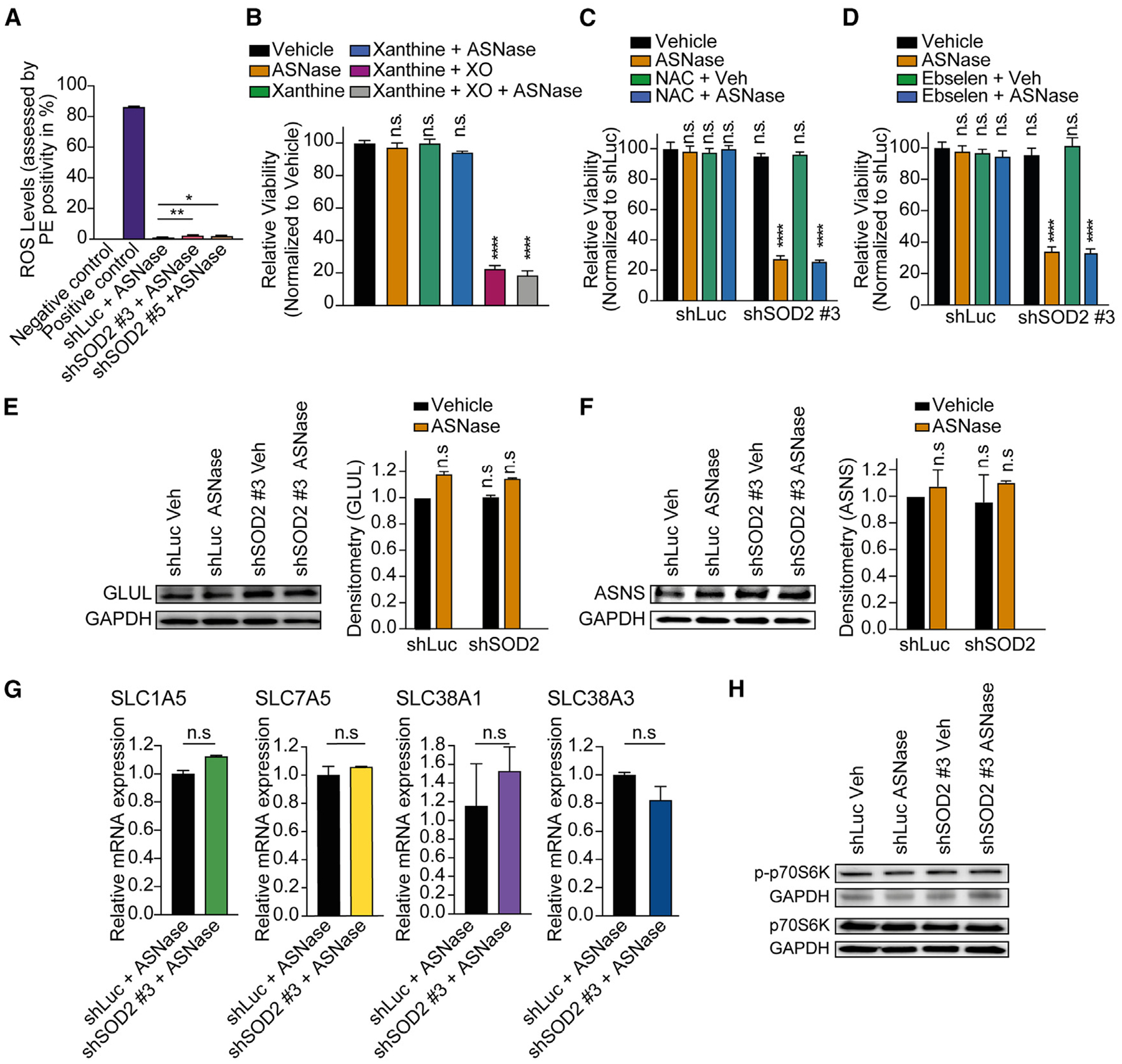
SOD2-regulated asparaginase response is independent of known SOD2-associated pathways (A) CCRF-CEM cells were transduced with the indicated shRNAs and treated with 100 U/L asparaginase. ROS levels were measured by assessing the percent of PE positivity with flow cytometry in biological duplicates. Antimycin A served as a positive control and N-acetylcysteine (NAC) as a negative control. (B) CCRF-CEM cells were treated with vehicle, 1 mM xanthine, 1 mM xanthine plus 0.01 U/mL xanthine oxidase (XO) in the presence or absence of 100 U/L asparaginase in biological triplicates. Relative viability was assessed after 8 days of treatment. Cell counts were normalized to vehicle-treated cells. (C) CCRF-CEM cells were transduced shLuc or shSOD2 #3 and treated with vehicle or 5 mM NAC in the presence or absence of 100 U/L asparaginase in biological triplicates. Relative viability was assessed after 8 days of treatment. Cell counts were normalized to shLuc-transduced, vehicle-treated cells. (D) CCRF-CEM cells were transduced with the indicated shRNA and treated with either vehicle or 20 μM ebselen in the presence or absence of 100 U/L asparaginase in biological triplicates. Relative viability was assessed as in (C). (E and F) Cells were transduced with shLuc or shSOD2 #3 and treated with vehicle or 100 U/L asparaginase. Protein levels of asparagine synthetase (ASNS), glutamine synthetase (GLUL), and GAPDH were assessed by western blot analysis. Densitometry was performed in biological duplicates for the target bands (ASNS or GLUL) and normalized to their respective GAPDH. (G) Cells transduced with shLuc or shSOD2 #3 were treated with 100 U/L asparaginase after confirmation of an efficient knockdown. Relative expression of amino acid transporters was assessed by RT-qPCR analysis in biological duplicates. (H) Cells transduced with shLuc or shSOD2 #3 were treated with vehicle or 100 U/L asparaginase after confirmation of an efficient knockdown. Protein levels of p-p70S6K, p70S6K, and GAPDH were evaluated by western blot analysis. All error bars represent SEM. ****p ≤ 0.0001, **p ≤ 0.01, *p < 0.05, n.s. *p* ≥ 0.05 by one-way ANOVA with Dunnett’s adjustment for multiple comparisons (A–F) and two-sided Student’s t test with Welch adjustment (G). See also Figures S2, S3, and Table S3.

Inhibition of SOD2 showed only a modest increase in ROS when compared with the Luciferase (Luc) control in the presence of asparaginase (Figure 3A), while the positive control antimycin A^29,30^ displayed a striking ROS generation. In line, treatment with exogenous superoxide mimetics xanthine or xanthine plus xanthine oxidase resulted in the previously reported baseline toxicity^27^ (Figure 3B), but did not induce an exacerbated cell death in response to asparagine depletion, despite showing the expected increase in ROS levels (Figure S2A).

In previous studies, inhibition of the SOD1 isoform has been reported to increase hydrogen peroxide levels in non-small lung cell carcinomas^27^ by diminishing the activity of glutathione peroxidase (GPX), an effect rescued by treatment with the ROS scavenger N-acetyl-cysteine (NAC), or the GPX mimetic ebselen.^27^ We thus wondered whether the inhibition of SOD2 displays a similar effect on GPX activity. However, treatment with NAC or ebselen was not able to block asparaginase-induced cell death upon SOD2 inhibition (Figures 3C and 3D). On balance, inhibition of SOD2 surprisingly appeared to regulate sensitization to asparagine starvation in a manner independent of ROS. Our findings that SOD2, but not SOD1 and SOD3, exhibited low baseline toxicity in the absence of asparaginase is in line with the observation that SOD2 inhibition does not dramatically change the ROS levels in cancer cells such as leukemia cells, which collectively suggests the activation of other redox pathways to compensate.^31,32^

We next wondered whether SOD2 inhibition affects the expression of relevant amino acid enzymes or transporters, but found no consistent effect on the expression of asparagine synthetase, glutamine synthetase (Figures 3E and 3F), or relevant amino acid transporters^33^ upon inhibition of SOD2 and asparaginase treatment (Figure 3G). In addition, asparagine depletion did not consistently affect expression levels of SOD2 in a panel of cancer cell lines as well as B-ALL PDXs (Figures S2B–S2C; Table S3).

To assess whether SOD2 is interconnected with mTORC1,^34,35^ we leveraged phosphorylation of p70S6K (Thr389) as an activity marker of mammalian target of rapamycin but could not find any remarkable changes in shSOD2 cells treated with asparaginase (Figure 3H).

In the regulation of metabolic pathways, SOD2 can be activated through deacetylation at amino acid position 68 (K68) by the mitochondrial sirtuin SIRT3,^36,37^ which can be up-regulated in response to calorie restriction as an effort to restore mitochondrial function under stress.^38^ However, assessment of SIRT3 expression in cells treated with asparaginase did not yield any significant differences (Figure S2D). In line, K68 levels of SOD2 did not show any remarkable differences in the presence of an asparagine depletion by contrast with the positive control nicotinamide^36^ (Figure S2E). Additionally, the knockdown of SIRT3 did not exacerbate asparaginase-induced cell death (Figure S2F).

Previous findings on SOD2-dependent cell-cycle fluctuations, particularly in the G1 phase,^39,40^ prompted us to check for cell-cycle-dependent changes in shSOD2 cells. However, we could not find any significant effects (Figures S3A–S3B), indicating the independence of previously characterized SOD2-related pathways.

### SOD2 promotes cell survival during amino acid starvation by interacting with UBR2

**Figure 4.**
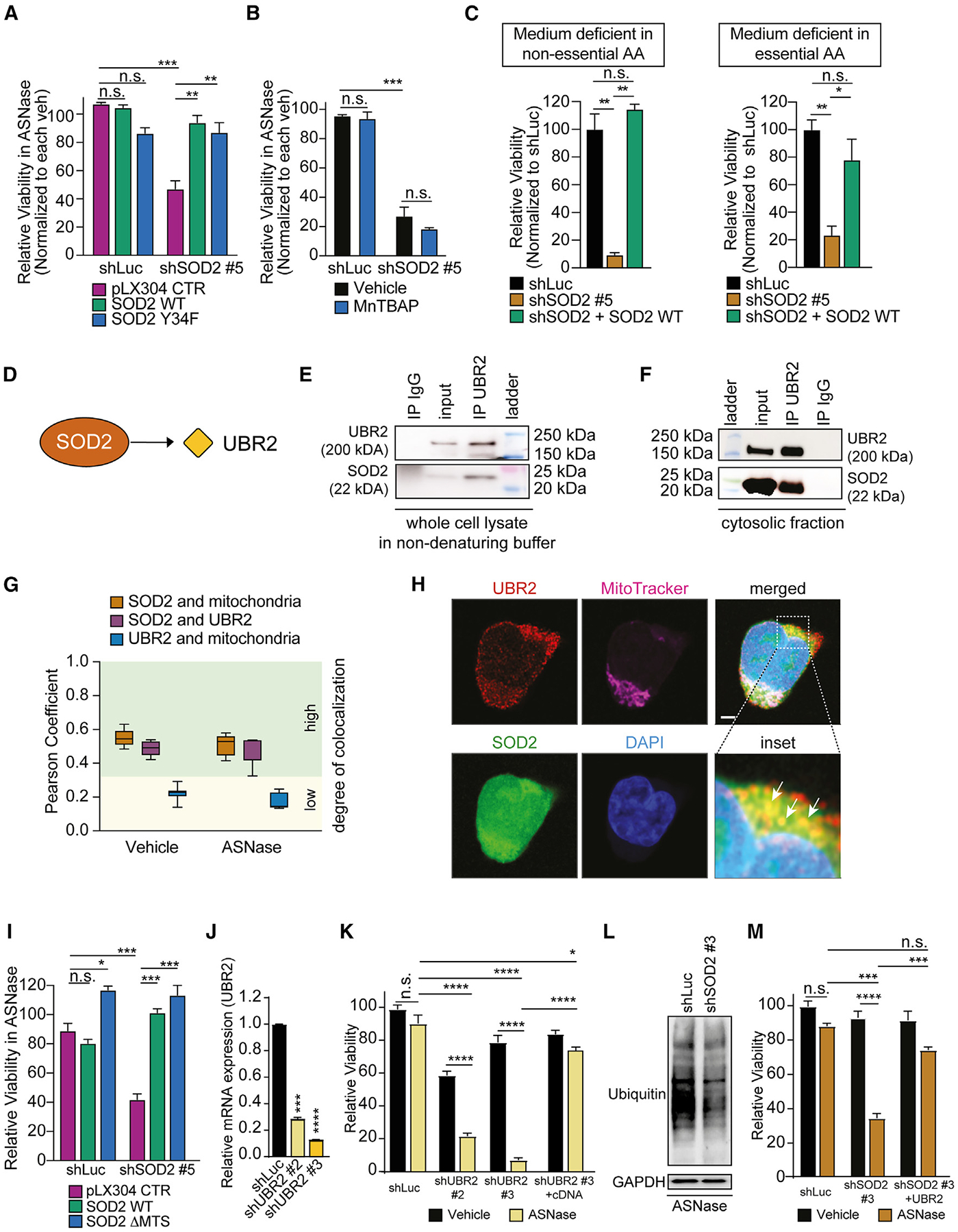
SOD2 promotes cell survival during amino acid starvation by interacting with UBR2 (A) Jurkat cells were transduced with shLuc, or shSOD2 #5, followed by transduction with a V5-tagged pLX304 control vector, pLX304-SOD2 wildtype (WT), or pLX304-SOD2 Y34F vector. After 4 days of treatment, viability was assessed in biological duplicates. Cell counts were normalized to vehicle-treated cells for each condition. (B) Jurkat cells were transduced with shLuc, or shSOD2 #5, and treated as indicated. After 8 days of treatment, viability was assessed. Cell counts were normalized to vehicle-treated cells. (C) Jurkat cells were transduced with the indicated shRNAs and pLX304-SOD2 WT vector and cultured in RPMI medium supplemented with only essential amino acids (EAA) or non-essential amino acids (NEAA) in biological duplicates. Cell counts were normalized to shLuc-transduced cells for each condition. (D) Schematic depiction of the interaction of SOD2 with UBR2. The protein-protein interaction is based on the BioPlex database.^44,45^ (E and F) Immunoprecipitation of UBR2 reveals a co-immunoprecipitation with SOD2 in Jurkat cells in a whole cell lysate using a non-denaturing buffer (E) and in a cytosolic cell fraction (F). (G) Jurkat cells were treated with vehicle or 100 U/L asparaginase for 48 h, and immunofluorescence staining was observed using a Zeiss LSM780 microscope. The co-localization of indicated targets was assessed by the Pearson correlation coefficient and ranked based on their degree of co-localization. (H) Representative images using super-resolution microscopy. Arrows indicate co-localization of UBR2 and SOD2. Scale bar, 2 μm (I) Jurkat cells were transduced with shLuc, or shSOD2 #5, followed by transduction with a V5-tagged pLX304 control vector, pLX304-SOD2 WT, or pLX304-SOD2 ΔMTS vector. After 8 days of treatment, viability was assessed in biological duplicates. Cell counts were normalized to vehicle-treated cells for each condition. (J) Jurkat cells were transduced with shUBR2 #2 and #3. Knockdown was confirmed by RT-qPCR in biological duplicates. (K) Cells were transduced with indicated shRNAs in the presence or absence of a UBR2 cDNA and treated with vehicle or 100 U/L asparaginase in biological triplicates. After 4 days of treatment, relative viability was assessed. Counts were normalized to shLuc-transduced cells. (L) Jurkat cells were treated as indicated. After a confirmed knockdown, cells were harvested, and protein levels of total ubiquitin and GAPDH were assessed by western blot analysis. (M) Jurkat cells were transduced with shLuc or shSOD2 and, subsequently UBR2 cDNA. After 4 days of treatment with vehicle or asparaginase (100 U/L) in biological duplicates, relative viability was assessed. Cell counts were normalized to shLuc-transduced, vehicle-treated cells. All error bars represent SEM. ****p ≤ 0.0001, ***p ≤ 0.001, **p ≤ 0.01, *p < 0.05, n.s., *p* ≥ 0.05 by one-way ANOVA with Dunnett’s adjustment for multiple comparisons (A–C, I–K, and M). See also Figure S4.

Next, we wondered whether SOD2-mediated sensitization to amino acid starvation is regulated by its dismutase activity. We, thus, leveraged the dismutase dead variant Y34F of SOD2,^41^ which fully phenocopied the effect of the SOD2 cDNA (Figure 4A and S4A). In addition, we failed to rescue shSOD2-mediated asparaginase sensitization by leveraging the SOD mimetic MnTBAP^42,43^ (Figures 4B and S4B). These data collectively indicate a dismutase-independent function of SOD2.

The observation that SOD2 inhibition sensitizes selectively to asparaginase out of all tested chemotherapeutic agents prompted us to ask whether SOD2 inhibition mediates cancer cell sensitization selectively to asparagine depletion, or whether it represents a broader cellular amino acid starvation phenotype. To test this, we cultured T-ALL cells transduced with shSOD2 in the absence of essential or non-essential amino acids, resulting in a striking sensitization, which could be rescued by the expression of SOD2 cDNA (Figure 4C).

To further dissect the mechanistic underpinnings of the SOD2-mediated phenotype, we leveraged the Bioplex Interactome database^44,45^ and identified the protein UBR2, a major E3 ubiquitin ligase, mainly associated with the Arg/N-degron protein degradation pathway,^46–48^ as a binding partner of SOD2 (Figure 4D). While UBR2 is localized in the cytosol, the previously described functions of SOD2 have been linked to its mitochondrial localization.^24^ Leveraging subcellular fractionation assays, we found that SOD2 is not only localized in the mitochondria but also the cytosol in different cancer cell line models (Figure S4C). Indeed, SOD2 and UBR2 co-immunoprecipitated in a whole cell lysate using a non-denaturing lysis buffer (Figure 4E), as well as in an isolated cytosolic cell fraction (Figures 4F and S4D). Of note, leveraging immunofluorescence super-resolution microscopy, we could also find co-localization of SOD2 and UBR2 (Figures 4G and 4H).

Next, we took advantage of a construct lacking the mitochondrial target sequence of SOD2 (ΔMTS-SOD2). Upon SOD2 depletion, this protein failed to translocate to the mitochondria by contrast with the SOD2 wildtype control (Figure S4E), but could still fully rescue the shSOD2-mediated sensitization phenotype (Figures 4I and S4F). These findings collectively add up to the line of evidence whereby the cytosolic population of SOD2 plays a crucial role in mediating the phenotype.

We then turned our attention to UBR2 as an interaction partner of SOD2. Intriguingly, the knockdown of UBR2 (Figure 4J) fully phenocopied the effect of SOD2 inhibition and resulted in profound asparaginase sensitization (Figure 4K), which could be rescued by expressing a UBR2 cDNA.

Assessing levels of total ubiquitinated proteins, we found decreased levels in both normal and asparagine-starved conditions (Figures 4L and S4G), which could not be rescued by providing an excess of asparagine in normal conditions. We thus hypothesized that the SOD2 phenotype is dependent on UBR2 function. In line with this hypothesis, overexpression of a UBR2 cDNA could rescue shSOD2-mediated asparaginase sensitization (Figure 4M).

Previous studies could show that, in cancer cells, UBR2 is up-regulated in response to cachectic stimuli, including proinflammatory cytokines.^49^ We thus asked whether asparagine starvation induces an upregulation of UBR2 expression levels but failed to observe this phenotype (Figure S4H). Similarly, the knockdown of SOD2 also did not affect the expression levels of UBR2 (Figure S4H). On balance, these findings are consistent with the hypothesis that SOD2 regulates cancer cell survival during amino acid starvation by its interaction with UBR2.

### UBR1 inhibition phenocopies the defective response to amino acid starvation

UBR1 and UBR2 are sequelogous, functionally overlapping, E3 ubiquitin ligases that have been previously shown to dominate the targeting of Arg/N-degron substrates, and thus mediate the bulk degradation of model substrates bearing Arg/N-degrons (reviewed by Varshavsky^50,51^). The Arg/N-degron pathway targets specific proteins for degradation through the 26S proteasome or the autophagy-lysosomal pathway by recognizing their specific N-terminal residues or their internal degrons.^15–19,50–54^ Depending on their destabilizing N-terminal residue, target proteins are classified as either tertiary, secondary, or primary, based on the serial modification steps required to be recognized by the N-recognins. Primary residues can be categorized as type 1 (basic) or type 2 (bulky hydrophobic) (reviewed by Tasaki et al.,^18^ Varshavsky,^50,51^ Timms and Koren,^52^ and Sriram et al.^55^). In mammals, the Arg/N-degron pathway is characterized by the ability of specific E3 ubiquitin ligases (N-recognins), including UBR1, UBR2, UBR4, and UBR5, as well as the p62 regulator of autophagy, to recognize these protein targets.^46,54–58^

**Figure 5.**
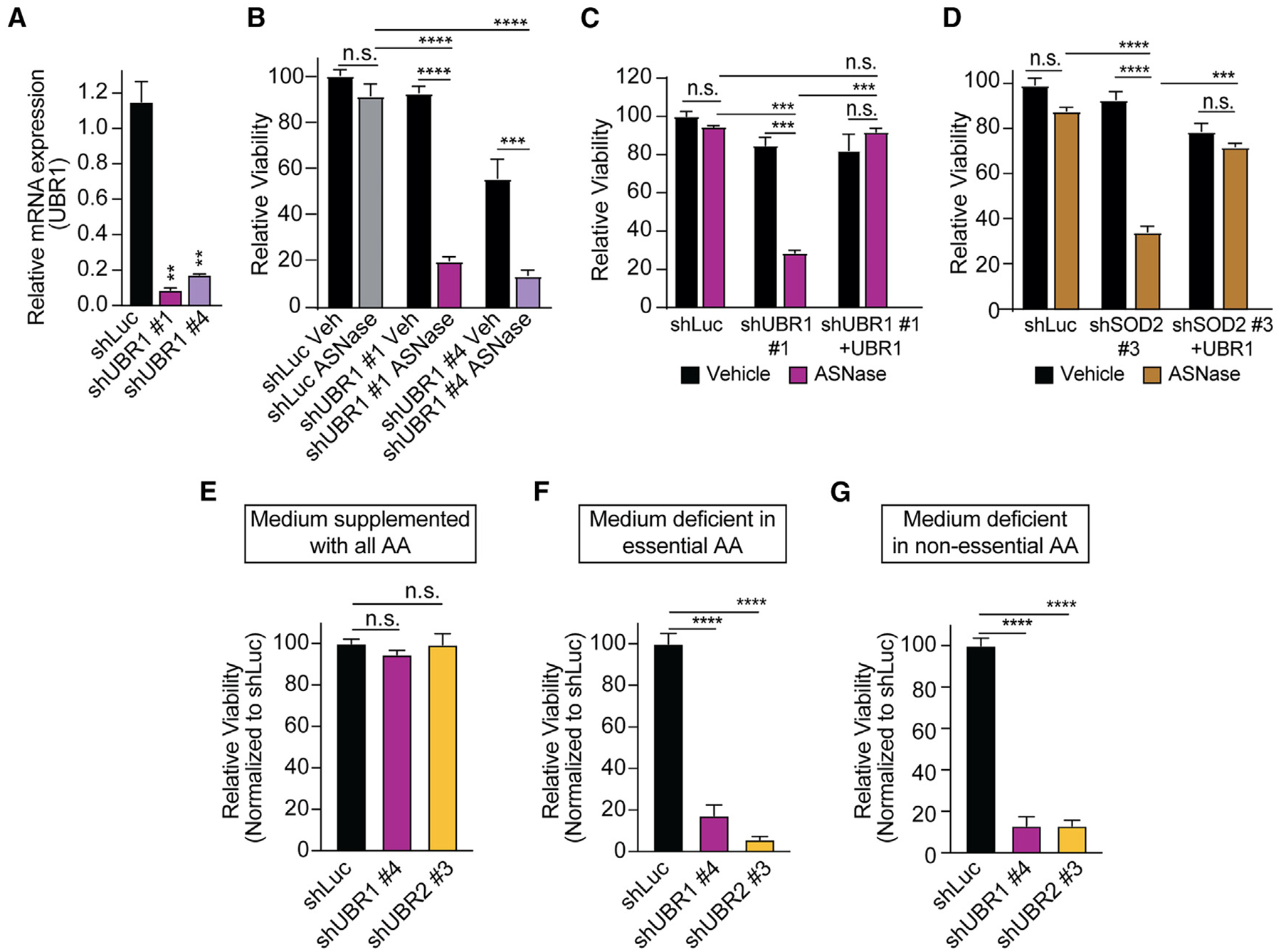
UBR1 inhibition phenocopies the defective response to amino acid starvation (A) Jurkat cells were transduced with shUBR1 #1 and #4. Knockdown was confirmed by RT-qPCR in biological duplicates. (B) Jurkat cells were transduced with shUBR1 and treated with vehicle or 100 U/L asparaginase in biological triplicates. After 4 days of treatment, viability was assessed and normalized to shLuc-transduced cells. (C) Jurkat cells were transduced with shLuc or shUBR1 in addition to a UBR1 cDNA. After 4 days of treatment with vehicle or asparaginase (100 U/L) in biological duplicates, relative viability was assessed. Cell counts were normalized to shLuc-transduced, vehicle-treated cells. (D) Jurkat cells were transduced with shLuc or shSOD2 and subsequently UBR1 cDNA. After 4 days of treatment with vehicle or asparaginase (100 U/L) in biological duplicates, relative viability was assessed as in (C). (E–G) Jurkat cells were transduced with indicated shRNAs and cultured in a medium supplemented with all amino acids, or in a medium deficient in essential or non-essential amino acids in biological triplicates. Cell counts were normalized to shLuc-transduced cells. All error bars represent SEM. ****p ≤ 0.0001, ***p ≤ 0.001, **p ≤ 0.01, n.s., *p* ≥ 0.05 by one-way ANOVA with Dunnett’s adjustment for multiple comparisons (A–G). See also Figure S5.

Thus, we next asked whether a knockdown of the sequelog UBR1 could phenocopy sensitization to amino acid starvation. Indeed, SOD2 and UBR1 co-immunoprecipitated (Figure S5A) and showed co-localization as assessed by immunofluorescence confocal microscopy (Figure S5B). The knockdown of UBR1 phenocopied the profound sensitization to asparagine depletion (Figures 5A and 5B), which could be rescued by overexpressing the UBR1 cDNA (Figure 5C). In line with these findings, overexpression of a UBR1 cDNA could rescue shSOD2-mediated asparaginase sensitization (Figure 5D). As for UBR2, we also did not observe any significant changes in UBR1 levels on mRNA or protein levels upon asparagine starvation (Figures S5C–S5D).

We then assessed whether UBRs promote survival in response to amino acid starvation. Knockdown of UBR1 or UBR2 did not impair the viability of T-ALL cells cultured in a medium supplemented with all amino acids (Figure 5E). However, in the absence of essential or non-essential amino acids, loss of UBR1 or UBR2 was highly toxic to these cells (Figures 5F and 5G).

### SOD2 is not a target of UBRs and does not appear to regulate the ATE1-dependent axis of the Arg/N-degron pathway

UBR1 and UBR2 are best known to function within the Arg/N-degron pathway. To investigate the role of SOD2 in this pathway and the directionality of the regulation between SOD2 and UBRs, we first wondered whether SOD2 is a substrate of the Arg/N-degron pathway.

**Figure 6.**
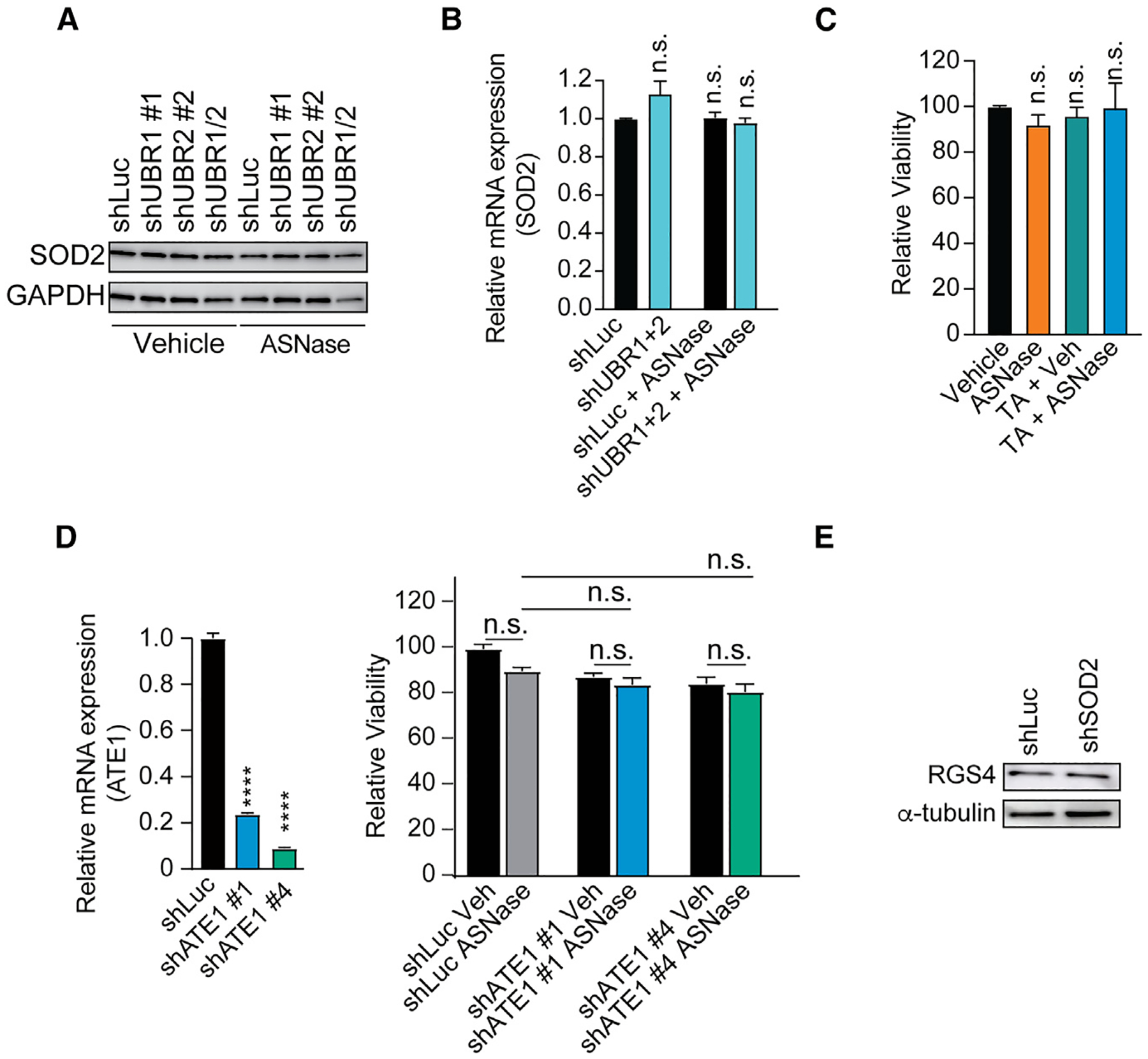
SOD2 is not a target of UBRs and does not appear to regulate the ATE1- dependent axis of the Arg/N-degron pathway (A) Jurkat cells were transduced with the indicated shRNA and treated with vehicle or asparaginase (100 U/L) for 48 h. SOD2 and GAPDH levels were assessed by western blot analysis. (B) Jurkat cells were transduced with the indicated constructs and treated with vehicle or asparaginase (100 U/L) for 48 h. SOD2 mRNA levels were assessed by RT-qPCR in biological duplicates and normalized to shLuc control cells. (C) Jurkat cells were treated with vehicle or 100 U/L asparaginase in the presence or absence of 1 μM tannic acid (TA). Relative viability was assessed after 4 days of treatment in biological duplicates. Cell counts were normalized to vehicle-treated cells. (D) Jurkat cells were transduced with indicated shRNAs, and knockdown efficiency was assessed by RT-qPCR in biological duplicates (left). Upon successful knockdown, cells were treated with vehicle or 100 U/L asparaginase (right). Relative viability was assessed after 4 days of treatment in biological triplicates. (E) Jurkat cells were transduced with indicated shRNAs. Levels of RGS4 and alpha-tubulin were assessed by western blot analysis. All error bars represent SEM. ****p ≤ 0.0001; n.s., *p* ≥ 0.05 by one-way ANOVA with Dunnett’s adjustment for multiple comparisons (B–E). See also Figure S5.

As previously described for the identification of N-degron substrates,^17,20^ we induced a knockdown of UBR1 and/or UBR2 (Figure S5E), but failed to see measurable differences in SOD2 protein expression on these experimental manipulations (Figure 6A). Previous studies identifying specific N-degrons could show that the Arg/N-degron pathway can also modulate the translation of the mRNAs.^17,20,54,59^ Thus, we also checked for changes in mRNA levels of SOD2 but could not detect any significant differences (Figure 6B), arguing against SOD2 as a substrate of the N-degron pathway.

If SOD2 is not a target of the UBRs, does SOD2 function upstream and stimulate protein degradation via the Arg/N-degron pathway? For this, we focused on the ATE1-dependent axis of protein degradation. The ATE1 Arg-tRNA protein transferase (arginyltransferase or R-transferase) conjugates Arg to secondary N-terminal residues, thus converting them to primary type 1 residues.^60,61^ Importantly, ATE1 seems to be solely responsible for the N-terminal arginylation.^62^ To dissect this axis, we treated leukemia cells with tannic acid, an inhibitor of ATE1 activity,^63^ but could not observe any asparaginase sensitization (Figure 6C). In line, the knockdown of ATE1 did not sensitize cells to asparaginase (Figure 6D).

To further strengthen the conclusion that asparaginase resistance is independent of ATE1, we next focused on the protein RGS4 as a known substrate downstream of the ATE1 axis.^17,64^ However, in line with the findings described above, the knockdown of SOD2 did not change levels of RGS4 (Figure 6E). Collectively, these findings suggest that SOD2 is not involved in the arginylation-dependent degradation of distinct substrates within the mammalian N-degron pathway. Additionally, inhibition of SOD2 led to a conceivable decrease in total ubiquitinated proteins upon asparagine starvation (Figure 4L). While the Arg/N-degron pathway has been demonstrated to catalyze most ubiquitin conjugation in muscle cells, it only makes a minor contribution to overall protein ubiquitination in cancer cells.^65^ On balance, these findings indicate that SOD2 does not appear to mediate the degradation of particular N-degron substrates.

### Inhibition of SOD2 impairs global cellular protein degradation upon starvation

**Figure 7.**
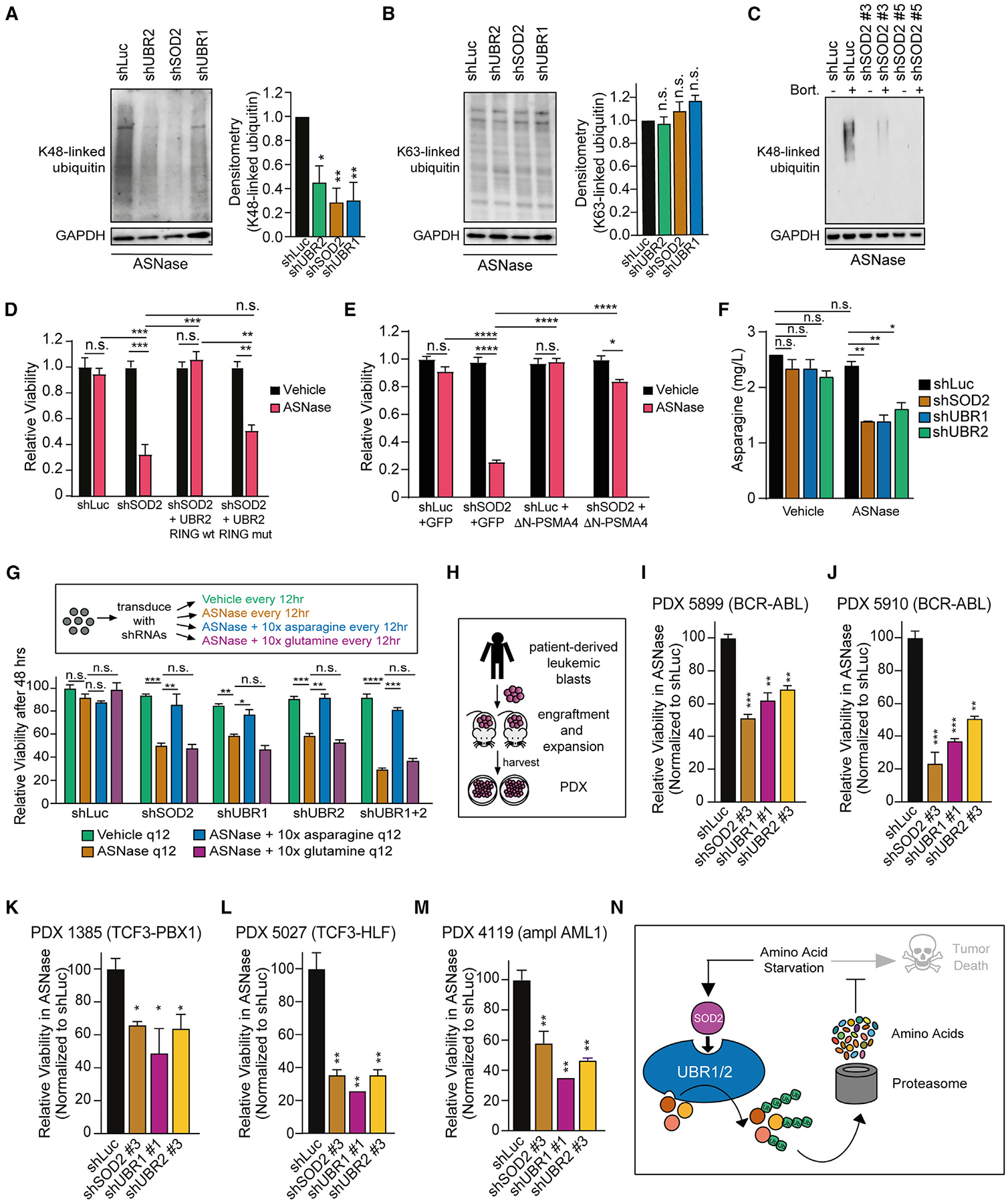
SOD2-mediated protein breakdown promotes cancer cell fitness upon amino acid starvation and reflects an adaptive proteasomal degradation machinery (A and B) Jurkat cells were transduced with the indicated shRNAs and treated with 100 U/L of asparaginase. Western blot analysis was performed for K48-linked ubiquitin (A) or K63-linked-ubiquitin (B) together with GAPDH. Densitometry analysis was performed for target protein levels and normalized to the respective GAPDH signal in biological triplicates. (C) Jurkat cells were transduced with the indicated constructs and treated with 1,000 U/L of asparaginase for 48 h in the presence or absence of bortezomib (20 nM for 5 h). Western blot analysis was performed for K48-linked ubiquitin. Note that a very short time of exposure was chosen due to the striking K48 accumulation in bortezomib-treated shLuc cells, resulting in a lack of signal in samples not treated with bortezomib. (D) Jurkat cells were transduced with shLuc or shSOD2 in addition to a pLX304-UBR2 RING wildtype (WT) or mutant (mut) vector, and viability was assessed after 4 days of treatment in biological duplicates. Viability was normalized to each vehicle condition. (E) Jurkat cells were transduced with shLuc or shSOD2 in addition to a pLX304-GFP control vector, or the hyperactive proteasomal subunit pLX304-ΔN-PSMA4. After 8 days of treatment with vehicle or asparaginase (100 U/L) in biological triplicates, relative viability was assessed. Cell counts were normalized to shLuc-transduced, vehicle-treated cells. (F) Jurkat cells were transduced as indicated and treated with vehicle or 10 U/L asparaginase for 48 h. Asparagine content in cells was quantified by liquid chromatography tandem mass spectrometry. (G) (Inset) Jurkat cells were first transduced with shLuc, shSOD2 #3, shUBR1 #1, shUBR2 #3, or a combination of shUBR1+2. Cells were then treated with vehicle, or asparaginase (100 U/L) in a complete growth medium (RPMI-1640 + 10% fetal bovine serum), or treated with asparaginase in a complete growth medium supplemented with 10× of L-asparagine or 10× of L-glutamine. Fifty percent of the media was removed every 12 h and replaced with fresh growth medium, supplemented with the appropriate concentration of asparaginase, asparagine, or glutamine. (Bottom) The viability was assessed after 48 h by counting viable cells and normalized to the viability in vehicle controls. (H) Schematic depiction of generation of B-ALL PDX models used in this study. (I–M) ALL PDX specimens were transduced with the indicated constructs, and treated with asparaginase (100 U/L) for 48 h. Viability was normalized to shLuc cells. Note that treatment could not be extended beyond 48 h due to the limited life span of PDX cells. (N) Proposed model. All error bars represent SEM. ****p ≤ 0.0001, ***p ≤ 0.001, **p ≤ 0.01, *p < 0.05, n.s., *p* ≥ 0.05 by one-way ANOVA with Dunnett’s adjustment for multiple comparisons (A, B, D–G, and I–M). See also Figure S5–S10.

Next, we set out to strengthen the hypothesis that SOD2 regulates bulk cellular protein degradation. If inhibition of SOD2 decreases global proteasomal protein degradation by interaction with the E3 ubiquitin ligases UBR1 and UBR2 upon amino acid starvation, then SOD2 inhibition should lead to a measurable decrease in K48-chain ubiquitinated proteins. Indeed, inhibition of SOD2 led to a significant reduction in total K48-chain ubiquitinated proteins in asparaginase-treated T-ALL cells, and this effect could be phenocopied by inhibition of the UBRs (Figure 7A). Reprogramming the ubiquitin chains from K48- to K63-linked ubiquitin can redirect proteins to the autophagy-lysosome system.^66,67^ However, levels of K63-linked ubiquitin remained unchanged upon inhibition of SOD2 or UBRs (Figure 7B). To further assess the role of autophagy-mediated protein degradation, we measured levels of the autophagy marker P62 (also known as SQSTM1) by western blot analysis. P62 is degraded by autophagy; thus, levels of this protein are inversely correlated with the rate of autophagy.^68^ However, we could not find any remarkable differences in P62 expression (Figure S5F). Moreover, treatment with inhibitors of lysosomal protein degradation, which blocks autophagy-induced protein degradation,^68^ had no effect on sensitivity to SOD2 inhibition and asparaginase (Figure S5G).

We then took advantage of the proteasomal inhibitor bortezomib, which has been demonstrated to decrease cellular proteasomal activity, marking its ability to impair the rate of global proteasomal protein degradation.^69–71^ We found that treatment with asparaginase and bortezomib led to a remarkable increase in K48-linked ubiquitin in shLuc control cells, an effect that was strikingly reduced upon inhibition of SOD2 (Figure 7C). These findings indicate that SOD2 is required to induce ubiquitination of target proteins. Additionally, proteasomal degradation was decreased upon inhibition of SOD2 and treatment with asparaginase (Figure S6A).

To support the hypothesis that the SOD2-mediated phenotype is dependent on protein ubiquitination through the UBRs, we expressed a RING domain wildtype as well as a RING domain defective variant of UBR2 (Figure S6B).^72^ Expression of the wild-type construct could fully restore viability in SOD2-deficient cells (Figure 7D), while expression of the defective variant failed to rescue the sensitization phenotype (Figure 7D).

Next, we set out to gain insights into the regulation of UBRs by SOD2. Ubiquitin ligases are not only functionally but also spatially linked to the proteasome, and their physical proximity allows for efficient substrate processing.^73–75^ Additionally, the proteasome can serve as a platform for E3 ligases to enhance substrate ubiquitination and degradation.^76^ We thus leveraged confocal microscopy and asked whether SOD2 regulates the physical proximity of UBRs with the proteasome, as assessed by immunofluorescence for the proteasomal subunit PSMA4. Indeed, UBR1 and UBR2 strongly co-localized with the proteasome, an effect that was markedly reduced upon knockdown of SOD2 (Figure S6C).

Does the inhibition of SOD2 mediate cancer cell sensitivity to amino acid starvation by inhibition of protein degradation? For this, we leveraged a hyperactive open-gate mutant of the proteasomal subunit PSMA4 (ΔN-PSMA4), which is sufficient to stimulate the degradation of a wide range of proteasomal substrates.^77^ Indeed, expression of the ΔN-PSMA4 proteasomal subunit fully blocked shSOD2-mediated sensitization to asparagine starvation (Figure 7E). These findings indicate that SOD2 inhibition sensitizes cancer cells to amino acid starvation by inhibiting proteasomal degradation of proteins.

We previously showed that proteasomal protein degradation dependent on GSK3α activity is a catabolic source of proteinogenic amino acids, which is blocked by WNT-dependent stabilization of proteins (WNT/STOP).^21,22^ To assess whether SOD2 is a regulator of WNT/STOP, we overexpressed the E3 ubiquitin ligase component FBXW7,^78–80^ which can rescue WNT/STOP-induced asparaginase hypersensitivity.^21,22^ As a control, we leveraged the FBXW7 R465C mutant, which displays impaired binding to its canonical phosphodegron.^81^ However, overexpression of FBXW7 wildtype failed to block shSOD2-mediated asparaginase hypersensitivity (Figure S7A), while it rescued shGSK3α-mediated asparaginase sensitization (Figure S7B). We note that mutations in FBXW7 are a common event, particularly in T-ALLs, and that Jurkat cells harbor an endogenous FBXW7 mutation.^82,83^ However, FBXW7 overexpression could also not reverse shSOD2-mediated asparaginase sensitization in KOPTK1 cells, which do not harbor identifiable FBXW7 mutations^82^ (Figures S7C).

Based on our previous model, asparaginase sensitization upon inhibition of GSK3α could be reversed by overexpressing ubiquitin ligases such as FBXW7. We thus reasoned that the overexpression of UBRs as E3 ubiquitin ligases should rescue shGSK3α-mediated sensitization if the SOD2-UBR axis functions within the same pathway. However, overexpression of UBR2 failed to compensate for shGSK3α-mediated asparaginase sensitization (Figure S7D).

To assess the role of other E3 ubiquitin ligases, we turned our attention to STUB1 (encoding for CHIP), a critical E3 ubiquitin ligase increasing cellular levels of K48-linked ubiquitinated proteins.^84–86^ Overexpression of STUB1 failed to rescue shSOD2-mediated sensitization by contrast to UBR1, or UBR2 (Figure S7E), underscoring the role of UBRs in the SOD2-dependent phenotype.

We could previously show that GSK3α undergoes supramolecular assembly with the ubiquitin-proteasome system in so-called GSK3α bodies to promote the degradation of proteins.^87^ Leveraging immunofluorescence confocal microscopy, we failed to identify UBR1 as a component of the GSK3α bodies (Figure S7F). In line, the knockdown of SOD2 or UBR1 did not impair the ability of GSK3α to undergo spatial sequestration (Figure S7G). Collectively, the described observations argue against the role of the SOD2-UBR axis in the WNT/STOP pathway.

### SOD2-mediated protein breakdown promotes cancer cell fitness upon amino acid starvation and reflects an adaptive proteasomal degradation machinery

Protein ubiquitination and proteasomal degradation are of high importance for the degradation of unfolded, or damaged proteins.^88^ Thus, we wondered whether inhibition of SOD2-mediated protein breakdown might promote cell death by activating the unfolded protein response (UPR). However, transduction of shRNAs targeting SOD2, UBR1, UBR2, or UBR1 and UBR2 and treatment with asparaginase did not induce an increase of the spliced XBP1 mRNA transcript, a well-known UPR marker^89^ (Figure S7H) by contrast with the thapsigargin-treated positive control.

Next, we applied unbiased mass spectrometry proteomics but found no individual proteins that were consistently up- or down-regulated upon inhibition of SOD2, UBR1, UBR2, or the combination of both (Figure S8; Table S4). These findings, together with the effects on K48-linked ubiquitin, and the reversal by direct stimulation of proteasomal activity, support a model in which SOD2-regulated response to amino acid starvation is mediated by the global effects on protein degradation.

This model suggests that leukemias resistant to asparaginase depend on the proteasomal breakdown of proteins, which provides amino acids,^90^ to sustain asparagine levels above a critical threshold during asparaginase treatment. This adaptive pathway can be disrupted by inhibiting SOD2. To test this, we performed amino acid quantification in cells depleted from SOD2, UBR1, or UBR2 in the absence or presence of asparaginase treatment. While the knockdown of these genes had little effect on asparagine concentrations in anabolic conditions, it significantly exacerbated the asparaginase-induced depletion of asparagine (Figure 7F), with no significant effect on other amino acids (Figures S9A–S9B).

We then assessed whether the depletion of asparagine is responsible for the cytotoxicity upon inhibition of SOD2 and asparaginase treatment. Intriguingly, shSOD2, or shUBR-induced sensitization to asparaginase, was fully blocked by providing a 10-fold excess of free asparagine, but not standard media or a 10-fold excess of glutamine, whose physiochemical properties resemble those of asparagine but that is not depleted upon asparaginase treatment^21,22^ (Figure 7G).

While our data show that SOD2-mediated proteasomal degradation plays a crucial role in promoting cancer cell fitness upon amino acid starvation, we next wondered whether this pathway also promotes survival in non-cancer cells. Of note, we previously showed that normal cells can compensate for adaptive protein degradation by relying on autophagy during longer periods of asparagine depletion.^22,87^ We thus turned our attention to cells derived from normal human intestinal epithelium (CCD841 cells).^91^ We focused on the requirement of SOD2-mediated protein degradation at the early stages of the amino acid response in these cells and could indeed observe a significant sensitization upon inhibition of SOD2 or UBRs (Figure S7I).

Our previous studies could demonstrate GSK3-dependent protein degradation as a key mechanism that allows drug-resistant cancer cells to tolerate asparagine starvation.^21,22,87^ However, resistance toward WNT/STOP-induced asparaginase sensitivity can occur,^87^ indicating that cells can activate alternative escape mechanisms to tolerate amino acid starvation.

To test the idea that SOD2-mediated proteasomal protein degradation reflects a compensatory mechanism for cancer cells, we generated WNT/STOP-resistant T-ALL single-cell clones. Of note, we could observe that shSOD2 could re-sensitize these clones (Figures S7J–S7K) demonstrating the essentiality of SOD2-mediated protein degradation as an independent proteasomal protein degradation pathway in maintaining cancer cell fitness upon starvation.

### Inhibition of SOD2 or UBRs sensitizes drug-resistant human leukemia PDXs to asparaginase

To test the pathophysiological relevance of our findings, we turned our attention to human PDX leukemia specimens, which were derived from patients with B-precursor acute lymphoblastic leukemia treated with asparaginase-intensive combination chemotherapy on contemporary clinical trials (Figure 7H; Table S3). The samples were collected at diagnosis or relapse from patients carrying unfavorable genetic alterations such as BCR-ABL or TCF3-HLF who went on to have poor treatment responses. In line with the clinical characteristics, the samples proved to be highly refractory to asparaginase treatment (Figures S10A–S10E).

Intriguingly, the knockdown of SOD2, or UBRs induced a striking asparaginase sensitization (Figures 7I–M) in the drug-resistant PDX cells with a comparable order of magnitude when compared with Jurkat T-ALL cells after 48 h of treatment (Figure S10F).

## DISCUSSION

Nutrient availability is fundamental to cellular homeostasis. However, the mechanisms that allow mammalian cells to tolerate amino acid starvation remain incompletely understood. The ubiquitin-proteasome machinery is responsible for the bulk of intracellular protein degradation in mammalian cells.^88,92^ Previous studies have implicated the role of proteasomal protein degradation in the maintenance of amino acid homeostasis. After acute deprivation of amino acids, proteasomal activity plays a crucial role in the maintenance of protein synthesis and cell survival.^9^ In addition, cell death in response to proteasome inhibition has been linked to a lethal amino acid shortage.^90^ While these studies provide a potential link between amino acid starvation and proteasomal activity, the regulation of the proteasomal protein degradation through the ubiquitin-proteasome system in response to amino acid deprivation is less well studied.

SOD2 is a member of the SOD family of antioxidants, which play an essential role in cellular protection against mitochondrial oxidative damage, while dismutase-independent functions are much less well studied. UBR1 and UBR2 are E3 ubiquitin ligases previously shown to function in the Arg/N-degron pathway,^50,51^ which facilitates the degradation of target substrates by recognizing destabilizing N-terminal amino acids. However, the function of UBRs distinct from their role in the Arg/N-degron pathway remains largely unknown. In this context, some studies could demonstrate that in yeast Ubr1 and Ubr2 function in a quality control pathway for the degradation of unfolded cytosolic proteins, a mechanism distinct from the N-degron pathway.^93–95^ While these studies indicate the role of UBRs in cellular protection against proteotoxic stress, their role in the metabolic stress response remains elusive.

The studies shown here demonstrate that SOD2 has a previously unrecognized dismutase-independent function to regulate global proteasomal protein degradation (Figure 7N). We propose that, in response to amino acid depletion, cancer cells can trigger protein degradation through the SOD2-UBR interaction as a catabolic source of amino acids to promote cell survival. Our model posits that asparaginase-resistant leukemias rely on proteasomal degradation of proteins, a catabolic source of amino acids,^90^ to maintain asparagine levels above a critical threshold during treatment with asparaginase, an adaptive pathway blocked by the inhibition of SOD2-dependent protein degradation. This reflects a cellular escape mechanism in cancer cells that heavily depend on proteasomal degradation to survive amino acid shortage, and that have developed independence from other proteolytic systems such as WNT/STOP. Thus, our findings highlight the possibility for cancer cells to opt for different proteasomal degradation routes as alternatives to supply amino acids. Of note, inhibition of SOD2 or UBRs highly sensitized drug-resistant human leukemia PDXs to asparaginase. Thus, our study emphasizes the importance of the development of inhibitors interfering with the SOD2-UBR interaction, which are expected to have a meaningful clinical benefit. Fully defining how SOD2 and UBRs molecularly interact, and how SOD2 signals the degradation of target proteins through the ubiquitin-proteasome system is thus of high interest for future investigation.

We note that UBRs, along with other genes that could be associated with asparaginase sensitization, such as GSK3A, did not score in our initial genome-wide screen. We believe this outcome highlights the limitations of guide RNA libraries, which tend to favor targeting early (5′-terminal) protein-coding exons rather than those that encode functional protein domains.

GSK3-dependent protein degradation was previously shown to provide a catabolic source of proteinogenic amino acids, which can be blocked by WNT/STOP.^21,22^ To date, regulators of WNT/STOP and associated ubiquitin ligases, as well as substrates, are not well characterized. Despite the role of SOD2-regulated protein degradation as a mechanism to generate an alternative supply of amino acids, the SOD2-UBR axis does not seem to be directly involved in WNT/STOP signaling. It is of high interest for future studies to characterize substrates of the SOD2-UBR axis and to investigate whether this axis shares protein targets for subsequent ubiquitination with distinct proteolytic systems, such as the Arg/N-degron pathway or the WNT/STOP pathway. A further intriguing question will also be whether these proteolytic pathways can substitute for each other under certain metabolic stress conditions.

Transduction of UBR1 and UBR2 was sufficient to rescue leukemia cells from shSOD2-mediated sensitization to asparaginase while transduction of FBXW7 or STUB1 could not reverse this effect. It is perhaps surprising that overexpression of selective E3 ubiquitin ligases targeting substrates for proteasomal degradation can suppress the effect of SOD2 depletion. However, the findings that specific ubiquitin ligases can rescue the effect on global proteasomal protein breakdown in the context of distinct cellular proteasomal degradation machineries is consistent with previous work,^79^ showing that a specific E3 ubiquitin ligase FBXW7 can rescue the effect of WNT/STOP, which impairs proteasomal breakdown of bulk cellular proteins. These findings collectively raise the possibility that specific E3 ubiquitin ligases can function as the principal E3 ubiquitin ligases in distinct cellular proteasomal degradation machineries. Distinguishing among these and other possibilities will require additional investigation.

While our findings indicate that activation of the UPR does not mediate cytotoxicity upon inhibition of SOD2-mediated protein breakdown, it does not exclude the possibility that binding of SOD2 to UBRs changes substrate specificity in the context of amino acid starvation to preferentially degrade unfolded, or misfolded proteins. Consequently, it will be of future interest to elucidate the physiological substrates of the SOD2-UBR axis in the context of cellular stress conditions and to fully characterize how SOD2 regulates protein ubiquitination and proteasomal degradation.

### Limitations of the study

While we find binding of UBR1 and UBR2 to SOD2, we observe a stronger binding affinity for UBR2. Despite being highly sequelogous proteins, previous studies could show differences in preferential binding of the two UBRs based on factors such as the dissociation constant of their binding protein^96^ or the electrostatic environment that may change the protonation state and/or conformation of proteins.^97–99^ This scenario may also apply to the interaction and affinity of SOD2 and UBR1/2, which will require an in-depth biochemical characterization. Thus, it will be of future interest to delineate which molecular factors determine the binding of SOD2 and the UBRs.

Additionally, SOD2-regulated adaptation to amino acid starvation through UBRs appears to be not mediated by the arginylation-dependent branch of the N-degron pathway, as demonstrated by the ATE1-independent phenotype (Figure 6). While this does not fully rule out the possibility of arginylation-independent branches of the Arg/N-degron pathway such as intracellular endopeptidases,^51,100,101^ bulk changes in total cellular ubiquitin levels (Figure 4L) are unlikely to be mediated through these branches, which rather target a subset of specific proteins for degradation.^17,20,59^

## STAR★METHODS

### EXPERIMENTAL MODEL AND STUDY PARTICIPANT DETAILS

#### Cell lines

HEK293T cells, T-ALL, AML, B-ALL, colorectal cancer cell lines, and normal colon cells (CCD841) cells were purchased from ATCC (Manassas, VA, USA) or DSMZ (Braunschweig, Germany), and cultured in RPMI-1640, MEM-alpha, Leibovitz’s L-15, or DMEM media (Thermo Fisher Scientific) with 10% or 20% fetal bovine serum (FBS, Thermo Fisher Scientific) and 1% penicillin/streptomycin (Thermo Fisher Scientific) at 37°C, 5% CO2. Cell lines used in these studies were not genotyped, however, exclusively early passages (max. 25) were used. Mycoplasma contamination was excluded using the MycoSpy Detection Kit according to the manufacturer’s instructions (Biontex Laboratories; most recently in December 2024). Further information on the characteristics of the cell lines can be found in Table S2.

#### Patient-derived xenografts (PDXs)

PDX cells used were from specimens obtained from the ALL-BFM 2000, 2009, or COALL 06–97 with informed consent and institutional review board approval in accordance with the Declaration of Helsinki. Patient-derived xenografts were generated by engraftment of viably frozen leukemic cells into immunodeficient mice, followed by harvesting and viably freezing, as described.^104^ PDX cells were cultured in MEM-alpha medium supplemented with 10% human serum (Sigma-Aldrich), 10% FBS (Sigma-Aldrich), 1% L-glutamine (Sigma-Aldrich), 1% penicillin/streptomycin (Thermo Fisher Scientific), 50 ng/mL human SCF (R&D systems), 20 ng/mL FLT3 (R&D systems), and 10 ng/mL human IL7 (R&D systems) at 37°C, 5% CO2. Further information on the characteristics of the PDXs can be found in Table S3.

### METHOD DETAILS

#### Lentiviral transduction and transient transfection

Lentiviruses were generated by co-transfecting plasmids of interest (pLKO.1, pLX304) together with packaging vectors psPAX2 (a gift from Didier Trono; addgene plasmid # 12260) and VSV.G (a gift from Tannishtha Reya; addgene plasmid # 14888) using OptiMEM (Thermo Fisher Scientific) and Polyethyleneimine (PEI, Carl Roth), as previously described.^21^ For concentrated virus, the virus-containing medium was ultracentrifuged at 24,000 rpm for 2 h at 4°C (Beckman Coulter), and the obtained pellet was resuspended in RPMI.

Lentiviral infections with the unconcentrated virus were performed by spinoculating cell lines with virus-containing media (1,500 g × 90 min) in the presence of 8 μg/mL polybrene (Merck Millipore). Selection with antibiotics was started 24 h after infection with neomycin (700 μg/mL for a minimum of 5 days; InvivoGen), puromycin (1 μg/mL for a minimum of 48 h; InvivoGen), or blasticidin (15 μg/mL for a minimum of 5 days; InvivoGen). Lentiviral infections with the concentrated virus were performed without spinoculation by directly adding the virus to the cultured cells. Antibiotic selections were performed as described above.

Transient transfection was performed using Lipofectamine 2000 reagent (Thermo Fisher Scientific). Briefly, 800,000 cells were seeded in 2 mL of growth medium in 24 well plates. Five μg plasmid of interest and 10 μL lipofectamine were mixed with 300 μL OptiMEM, incubated for 10 min, and added to the wells. Antibiotic selection was begun after 48 h of incubation.

#### shRNA and expression plasmids

The following lentiviral shRNA vectors in pLKO.1 with puromycin were generated by the RNAi Consortium library and obtained from Sigma-Aldrich as bacterial stocks. Alternatively, oligos were purchased from Eurofins or IDT and cloned in pLKO.1 with puromycin or blasticidin (a gift from Bob Weinberg, addgene plasmid #8453, or Keith Mostov, addgene plasmid #26655, respectively). The shRNAs sequences are as follows: shLuciferase (TRCN0000072243), shSOD1#2 (TRCN0000018344), shSOD1#4 (TRCN0000039812), shSOD2#3 (TRCN0000005942), shSOD2#5 (TRCN0000005939), shSOD3 #3 (TRCN0000049077), shUBR1 #1 (TRCN0000003423), shUBR1 #4 (TRCN0000003424), shATE1#1 (TRCN0000034669), shATE1#4 (TRCN0000034672), shSIRT3 #1 (TRCN0000038889), shSIRT3 #2 (TRCN0000038890), and shGSK3α#1 (TRCN0000010340). shUBR2 #2 sequence, 5′-CCAATGGAATGGTACCTTT-3′ and shUBR2 #3 sequence, 5′- GCTGCTTCCTCCAAGAAAT-3′, were based on previous publications.^102,103^ SOD2 cDNA (NM_00 0636) was obtained from OriGene as a human-tagged cDNA clone RC202330 (Herford, Germany), or cloned in pLX304.

DNA constructs were designed with attB sites for gateway cloning and subsequently cloned into the pLX304 destination vector (a gift from David Root; addgene plasmid #25890). For the SOD2 dismutase dead variant, amino acid position 34 was mutated from Tyrosine to Phenylalanine, resulting in the Y34F mutant.^41^ For the ΔMTS-SOD2 construct, the first 18 amino acids (corresponding to the mitochondrial targeting sequence,^105^ https://csb-imlp.bio.rptu.de/) were deleted. Amino acid numbering was based on Uniprot ID P04179.

A hyperactive open-gate mutant of the human proteasomal subunit PSMA4, termed ΔN-PSMA4, was designed by deleting the cDNA sequences encoding amino acids 2 to 10 (SRRYDSRTT) of PSMA4 isoform NP_002780.1 (encoded by the transcript NM_002789.6), based on the data of Choi and colleagues.^77^ This ΔN-PSMA4 coding sequence was synthesized by gene synthesis and cloned into the pLX304 lentiviral expression vector in-frame with the C-terminal V5 tag provided by this vector, by GeneCopoeia (Rockville, MD).

Expression constructs expressing wild-type FBXW7 (also known as CDC4) or its R465C mutant were a gift from Bert Vogelstein (addgene plasmid #16652 and #16653). For lentiviral transduction, sequences encoding wild-type FBXW7, or the R465C were cloned into the pLX304 lentiviral expression vector by GeneCopoeia (Rockville, MD).

The RING wildtype and dead domains of UBR1 and UBR2 were designed based on the sequence from.^72^ RING dead domains were designed by replacing cysteine residues with alanine residues.^106^ For lentiviral transduction, sequences encoding the wild-type RING domain or the RING dead domain were cloned into the pLX304 or pLX302 lentiviral expression vector by Twist Bioscience (San Francisco, CA).

#### Assessment of drug response and apoptosis

Leukemia cells (25.000 per well) were seeded in 100 μL of complete growth medium in 96-well plates and incubated with indicated chemotherapeutic agents or vehicle. Cells were split every 48 h. Briefly, 20 μL of cells were mixed with 80 μL fresh culture medium, supplemented with vehicle or chemotherapeutic drugs at the indicated doses.

For the treatment of colorectal cancer cells, 150.000 cells were seeded in 2.5 mL of culture medium supplemented with the indicated drugs in a 12-well or 6-well plate format, respectively.

Cell viability was assessed by counting viable cells based on trypan blue vital dye staining (Thermo Fisher Scientific), according to the manufacturer’s instructions.

All asparaginase experiments were performed using pegaspargase (Oncaspar, Shire Pharmaceuticals, Lexington, MA), an FDA-approved PEGylated form of E. coli asparaginase. Other leveraged drugs include dexamethasone (Sigma-Aldrich), vincristine (Selleckchem), doxorubicin (Sigma-Aldrich), 6-mercaptopurine (Abcam), N-acetyl-cysteine (Sigma-Aldrich), xanthine (Sigma-Aldrich), xanthine oxidase (Sigma-Aldrich), MnTBAP (Sigma-Aldrich), tannic acid (Sigma-Aldrich), ebselen (Sigma-Aldrich), nicotinamide (Sigma-Aldrich), thapsigargin (Sigma-Aldrich) bortezomib (Sigma-Aldrich), bafilomycin (Sigma-Aldrich), ammonium chloride (Sigma-Aldrich), and chloroquine (Sigma-Aldrich). All drugs and reagents were used at the indicated concentrations.

Caspase 3/7 activity was assessed using the Caspase Glo 3/7 Assay (Promega) according to the manufacturer’s instructions.

#### Assessment of patient-derived xenografts

For assessment of mRNA levels, patient-derived xenografts (PDX) cells were seeded in 500 μL of the complete MEM-alpha growth medium and treated with vehicle or 100 U/L asparaginase for 48 h. For drug treatments, 40,000 PDX cells were seeded in 100 μL of the complete MEM-alpha growth medium and treated with vehicle or 100 U/L asparaginase for 48 h.

#### Single cell cloning

To derive single-cell clones, a limiting dilution strategy was used. Viable T-ALL cells were counted based on trypan blue and then diluted in RPMI (80 cells in 10 mL medium). Cells were seeded in 100 μL in a 96-well format and incubated at 37°C, 5% CO2. Plates were screened weekly for the formation of colonies. Established colonies were further expanded, and cultured for downstream experiments. Assessment of treatment response was performed as described above.

#### Quantitative Reverse Transcriptase PCR

RNA was isolated using the RNeasy kit (Qiagen) or NucleoZOL (Macherey-Nagel), and cDNA was made using the RevertAid Reverse Transcriptase (Thermo Fisher Scientific), Oligo(dT)18 Primer (Thermo Fisher Scientific), and dNTP mix (Biozym), or SuperScript VILO Master Mix (Thermo Fisher Scientific) according to the manufacturer’s protocol. RT-qPCR was performed using iTaq Universal SYBR Green Supermix (Bio-Rad) and QuantStudio 1 Real-Time PCR system (Applied Biosystems). The primers used are as follows: ATE1 (forward 5′-AGACCGAGGATGGCGAAGAAGT-3’; reverse 5′-AAGGCTGAAATTGTAAAGGTCGGCA-3′), beta-actin (forward 5′-CTGGCACCCAGCACAATG-3’; reverse 5′-GCCGATCCACACGGAGTACT-3′), GAPDH (forward 5′-TGGGGAAGGTGAAGGTCGGAGT-3’; reverse 5′-TGGAGGGATCTCGCTCCTGGAA-3′), SIRT3 (forward 5′-GGGCAGCAGCTCCCAGTTTCT-3’; reverse 5′-CCACTCCCCGGCGATCTGAAG-3′), SOD1 (forward 5′-CCGCACACTGGTGGTCCATGA-3’; reverse 5′-CAAGCCAAACGACTTCCAGCGT-3′), SOD2 (forward 5′-GCGTTGGCCAAGGGAGATGT-3’; reverse 5′-AGTCACGTTTGATGGCTTCCAGC-3′), SOD3 (forward 5′-TTGGAGGAGCTGGAAAGGTGCC-3’; reverse 5′-CCGCCGAGTCAGAGTTGGGC-3′), UBR1 (forward 5′-GGTGGTGTGCAGATCCTGCCT-3’; reverse 5′-GCAGACCGTGGGCACCTGAAA-3′), UBR2 (forward 5′-GAGGAGATTGCGGGGAAATGGC-3’; reverse 5′-GGTTGGG ACCCCTGCAGTAGATT-3′), SLC1A5 (forward 5′-CAGGCGGCTACTGCGGTT-3’; reverse 5′-GTTGGAAGGGAAGATATTTCTCGC A-3′), SLC7A5 (forward 5′-GGACCATTATCGGCTCGGGCAT-3’; reverse 5′-GATTGGACACATCACCCTTCCCG-3′), SLC38A1 (forward 5′-TGCAATCGGCCCGGAAAGGA-3’; reverse 5′-AAAATGGAAGCTTGACACCCCTGT-3′), SLC38A3 (forward 5′-CAACCGCG AGGCCAGACATC-3’; reverse 5′-AAGGCGCCTCCATGGCTCAG-3′), XBP1_spliced (forward 5′-TGCTGAGTCCGCAGCAGGTG-3’; reverse 5′-GCTGGCAGGCTCTGGGGAAG −3′).

#### Western Blot and dot blot analysis

Cells were lysed in Frackelton, or RIPA buffer (Merck Millipore) supplemented with cOmplete protease inhibitor (Roche) and PhosSTOP phosphatase inhibitor (Roche). Laemmli sample buffer (Bio-Rad), and β-mercaptoethanol (Sigma-Aldrich) were mixed with protein lysate before being run on a 4%–12% bis-tris polyacrylamide gel (Bio-Rad). Blots were transferred to polyvinylidene difluoride (PVDF) membrane (Carl Roth) and blocked with 5% BSA (AppliChem), or 5% sure block (LubioScience) in PBS with 0.1% Tween, and probed with the following antibodies: Phospho-p70S6Kinase (1:1000, Cell Signaling #9205, RRID: AB_330944), ASNS (1:1000, Santa Cruz #sc-376151, RRID: AB_11012145), GLUL (1:1000, Cell Signaling #80636, RRID: AB_2799956), alpha Tubulin (1:1000, Santa Cruz #sc-5286, RRID: AB_628411), SOD2 (1:1000, Thermo Fisher Scientific #MA1–106, RRID: AB_2536812), SOD2 (1:1000, Cell Signaling #13141S, RRID: AB_2636921) RGS4 (1:1000, Santa Cruz #sc-398348), UBR2 (1:1000, Abcam #ab217069), total ubiquitin (1:1000, Abcam #ab7254, RRID: AB_305802), K48-linked ubiquitin (1:1000, Millipore, #05–1307, RRID: AB_11213655), K63-linked ubiquitin (1:1000, Cell Signaling, Cat#5621, RRID: AB_10827985), SOD2-K68 (1:1000, Abcam #ab137037, RRID: AB_2784527), V5 tag (1:1000, Cell Signaling #13202, RRID: AB_2687461), P62/SQSTM1 (1:1000, Cell Signaling, Cat#5114, RRID: AB_10624872), ATP5A (1:1000, Santa Cruz, Cat#sc-136178, RRID: AB_2061764), GAPDH (1:1000, Santa Cruz #sc-365062, RRID: AB_10847862).

Detection of horseradish peroxidase–linked secondary antibodies (mouse and rabbit) with horseradish peroxidase substrate (Santa Cruz #sc-516102, RRID: AB_2687626 and #sc-2357, RRID: AB_628497) was visualized using Amersham Imager 800 (Cytiva). Densitometry analyses were performed by equalizing the signal-to-background ratio of each membrane and measuring the densitometry using Cytiva ImageQuant Software. For dot blot analyses, 3 μL of cell lysates of equal concentration were dropped onto a 0.2 μM nitrocellulose membrane (Bio-Rad) and dried for 30 min. The membrane was then blocked for 1 h with 5% BSA (AppliChem) in PBS and probed with the indicated antibodies.

#### Co-immunoprecipitation analysis

Briefly, cells were lysed in 300 μL Frackelton buffer (10 mM Tris-HCl (Carl Roth), 50 mM NaCl (Carl Roth), 30 mM NaPPi (Sigma-Aldrich), 50 mM NaF (Carl Roth), 1% Triton X-100 (Carl Roth)), supplemented with cOmplete protease inhibitor (Roche), and incubated for 30 min (4°C, rotating). Samples were then centrifuged (13.000 rpm × 12 min). Subsequently, 200 μL of PBS were added. The obtained supernatant was precleared with magnetic beads, incubated, and rotated at 4°C for 60 min. Beads were removed, and the supernatant was incubated with antibodies overnight (4°C, rotating). Magnetic beads were added and incubated (30 min at room temperature, 60 min at 4°C), followed by one washing step with Frackelton buffer and two washing steps with PBS. Washed beads were mixed with 1x Laemmli sample buffer (Bio-Rad), supplemented 100 mM DTT (PanReac Applichem), and incubated at 90°C for 5 min. Upon magnetic separation, the obtained supernatant was directly loaded on the 4–20% Mini-PROTEAN TGX Precast Protein Gels (Bio-Rad).

#### Subcellular fractionation

For the fractionation, cells (5 million) were pelleted, washed with PBS, and subsequently resuspended in 500μL of fractionation buffer (20mM HEPES pH7.4, 10mM KCl, 2mM MgCl_2_, 1mM EDTA, 1mM EGTA, 1mM DTT) supplemented with cOmplete protease inhibitor (Roche), and incubated on ice for 15 min. The cell suspension was then passed through a 27-gauge needle ten times, and incubated on ice for 20 min, followed by centrifugation at 720 × g for 5 min. The resulting supernatant containing cytoplasm, membrane, and mitochondria was further processed by centrifugation at 10,000 × g for 5 min. The supernatant (cytoplasm and membrane) was kept on ice while the resulting pellet containing the mitochondrial fraction was resuspended in 200μL TBS/0.1% SDS (Carl Roth) and briefly sonicated three times.

For the full lysates, cells (1.5 million) were resuspended in 100 μL RIPA buffer (Merck Millipore) supplemented with cOmplete protease inhibitor (Roche), incubated on ice for 20 min, followed by centrifugation at 20.000*g* × 10 min. The obtained supernatant was further processed as the full lysate. Samples to be analyzed were mixed with Laemmli sample buffer (Bio-Rad) before being run on a 4%–12% bis-tris polyacrylamide gel (Bio-Rad), as described above. For the subcellular fractionations shown in Figures S4C–S4E, 2 μL (~2%) of the full lysates, 35 μL (~7%) of the cytosolic and 35 μL (~18%) of the mitochondrial fractions were loaded.

#### Amino acid starvation assay

Cells (250,000) were cultured in 1 mL of amino acid free RPMI (US Bio) supplemented with either all non-essential amino acids: L-Alanine (Sigma-Aldrich, Cat#A7469), L-Arginine (Sigma-Aldrich, Cat#A5131), L-Asparagine (Sigma-Aldrich, Cat#A0884), L-Aspartic acid (Sigma-Aldrich, Cat#A9256), L-Cystine 2HCl (Sigma-Aldrich, Cat#30200), L-Glutamic Acid (Sigma-Aldrich, Cat#G1251), L-Glutamine (Sigma-Aldrich, Cat#G3126), Glycine (Sigma-Aldrich, Cat#G7126), L-Hydroxyproline (Sigma-Aldrich, Cat#H5534), L-Proline (Sigma-Aldrich, Cat#P0380), L-Serine (Sigma-Aldrich, Cat#S4500), L-Tyrosine disodium salt dihydrate (Sigma-Aldrich, Cat#T3754), or with all essential amino acids: L-Histidine (Sigma-Aldrich, Cat#H8000), L-Isoleucine (Sigma-Aldrich, Cat#I2752), L-Leucine (Sigma-Aldrich, Cat#L8000), L-Lysine hydrochloride (Sigma-Aldrich, Cat#L5626), L-Methionine (Sigma-Aldrich, Cat#M9625), L-Phenylalanine (Sigma-Aldrich, Cat#P2126), L-Threonine (Sigma-Aldrich, Cat#T8625), L-Tryptophan (Sigma-Aldrich, Cat#T0254), L-Valine (Sigma-Aldrich, Cat#V0500), or with all amino acids. For the amino acid add-back experiments, amino acid concentrations were used as in the formulation of regular RPMI-1640 (Thermo Fisher Scientific, Cat#11875119).

#### ROS measurement assay

Cells were cultured as described above and then harvested by centrifugation (500 g × 5 min). The supernatant was removed, and cells were further processed with the Dihydroethidium (DHE) Assay Kit (Abcam) according to the manufacturer’s protocol. Samples were subsequently analyzed using flow cytometry.

#### Cell cycle analysis

For cell cycle analyses, cells were cultured as described above and then harvested by centrifugation (500 g × 5 min). The supernatant was removed, and cell pellets were fixed with 4% paraformaldehyde (Alfa Aesar) for 10 min at room temperature and subsequently washed with PBS. Staining was performed with FxCycle PI/RNase Staining Solution (Thermo Fisher Scientific) according to the manufacturer’s protocol. Samples were analyzed by flow cytometry using a BD FACS Canto instrument. For assessment of cell size, cells were cultured in the presence of indicated reagents, and the forward scatter was subsequently determined by flow cytometry.

#### Immunofluorescence confocal microscopy

For immunofluorescence experiments, cells (400,000) were seeded in 1 mL of complete growth medium and incubated with appropriate drugs or vehicle. After 48 h, cells were harvested and fixed using 4% paraformaldehyde for 15 min at room temperature. Immunostaining was performed as previously described.^65^ Briefly, cells were washed twice with PBS, and permeabilized using 1% Triton X-100 (Carl Roth) for 15 min at room temperature, again followed by 2 wash steps (PBS). For immunostaining, cells were blocked in blocking buffer (5% goat serum and 5% BSA in PBS) for 45 min at room temperature. Subsequently, cells were incubated in a blocking buffer containing the primary antibody mouse anti-UBR1 (Santa Cruz sc-515753, 1:100), rabbit anti-GSK3α (Cell Signaling (CST) Cat#4818, RRID: AB_10831511, 1:100), rabbit anti-UBR2 (Abcam Cat#ab217069, 1:100), mouse anti-SOD2 (Thermo Fisher Scientific, Cat#MA1–106, RRID: AB_2536812, 1:200), rabbit anti-UBR1 (Abcam, Cat#138267, 1:100), or mouse anti-PSMA4 (Santa Cruz, Cat#sc-271297, RRID: AB_10608330). After 45 min, cells were washed twice with PBS and incubated in blocking buffer supplemented with the secondary antibody anti-mouse Alexa Fluor 488 (Thermo Fisher Scientific, A-11001, RRID: AB_2534069, 1:100), anti-mouse Alexa Fluor 555 (Thermo Fisher Scientific, A-21422, RRID: AB_2535844, 1:100), anti-rabbit Alexa Fluor 488 (Thermo Fisher Scientific, A-11008, RRID: AB_143165, 1:100), anti-rabbit Alexa Fluor 555 (Thermo Fisher Scientific, A-21428, RRID: AB_2535849, 1:100), or Alexa Fluor 647 (Thermo Fisher Scientific, A-21245, RRID: AB_2535813, 1:100) Cells were again washed twice and mounted on microscope slides using mounting media with DAPI or with separate DAPI to stain nuclei (ProLong Diamond Antifade Mountant with DAPI, Thermo Fisher Scientific; ProLong Gold Antifade Mountant, Thermo Fisher Scientific; DAPI, Carl Roth). Immunostaining was observed and imaged using a Zeiss LSM780 confocal microscope using the 63X objective. Representative super-resolution images were taken on an LSM900 Airyscan2.

Pearson’s correlation coefficient was calculated using custom-written MATLAB scripts. The analysis strategy follows the colocalization theory of Scientific Volume Imaging^107^ or https://svi.nl/ColocalizationTheory. Negative control for the correlation coefficient was obtained for each image by rotation of the second channel by 90° and 50% pixel shift, followed by the same correlation analysis as for the original image. The cut-off for a low degree of colocalization was set to a Pearson correlation coefficient of 0.3, as previously described.^108^

#### Mitochondrial staining and imaging

Cells were treated as described above. After 48 h, cells were incubated with MitoTracker Red CMXRos (Thermo Fisher Scientific) based on the manufacturer’s instruction using a 300 nM final working concentration of the MitoTracker. Cells were then fixed and permeabilized based on the manufacturer’s instructions, followed by confocal slide preparation as described above.

#### Amino acid quantification

Jurkat cells (400,000 per well) were transduced with shLuc, shSOD2, shUBR1, or shUBR2 and seeded in 1 mL of complete growth medium in a 24-well format. The growth medium was supplemented with final concentrations of 10 U/L asparaginase or vehicle. After 48 h of treatment, amino acid quantification of cells was performed as previously described.^22^ The entire amino acid profile was determined using LC/MS-MS. Values of ≤1.0 were set to 1.0.

#### Mass spectrometry analysis

Protein extracts were mixed with Laemmli buffer and incubated for 5 min at 95°C. Proteins were then alkylated by the addition of acrylamide up to a concentration of 2%, and incubated at room temperature for 30 min. SDS PAGE was performed on 12% gels in a mini protean cell (Bio-Rad). After electrophoresis, proteins were stained with GelCode Blue Safe Protein Stain (Thermo Fisher Scientific) for 15 min, and background staining was reduced with water. Each lane was cut into four pieces, which were further minced into 1 mm^3^ gel pieces. Further sample processing was done as previously described.^109^

Peptide samples were analyzed by a shot-gun approach in an LC-MS system (RSLC, Orbitrap Exploris 240, both Thermo Fisher Scientific) as described recently.^110^ Raw MS data were processed using Max Quant (version 2.0, Cox and Mann 2008), Perseus software (version 2.0.6.0),^111^ and human entries of Uniprot DB. Proteins were identified by a false discovery rate of 0.01 on protein and peptide levels.

Data normalization and statistical analysis were performed using R version 4.3.2 and Rstudio version 2023.09.0 + 463. The initially identified proteins were filtered for quantification. Only proteins detected in at least 70% of all samples were used. Missing values were imputed with a downshift of 1.8 and a width of 0.3. To offset differences in lane loading, quantile normalization was performed as described by.^112^ Additional normalization was computed using treatment cell counts of each condition. For the statistical analysis, limma functions, voom, and eBayes (trend = TRUE, robust = TRUE) were used to calculate and retrieve log-fold change and *p*-value.^113^

### QUANTIFICATION AND STATISTICAL ANALYSIS

Statistical analyses were performed using GraphPad Prism 9, or R package in the case of mass spectrometry analyses as described in detail for this experimental method. The number of biological replicates, the statistical test for each individual experiment and the precision measures are indicated in the figure legends. Statistical significance was defined as *p < 0.05 and is indicated in the figure legends.

## Supplementary Material

1

2

3

4

5

6

## Figures and Tables

**Table:** KEY RESOURCES TABLE

REAGENT or RESOURCE	SOURCE	IDENTIFIER
Antibodies		
mouse anti-GAPDH	Santa Cruz	Cat#sc-365062, RRID:AB_10847862
mouse anti-alpha tubulin	Santa Cruz	Cat#sc-5286, RRID: AB_628411
rabbit anti-Ubiquitin	Abcam	Cat#ab7254, RRID: AB_305802
rabbit anti-K48 linked ubiquitin	Millipore	Cat#05–1307, RRID: AB_11213655
rabbit anti-K63 linked ubiquitin	Cell Signaling	Cat#5621, RRID: AB_10827985
mouse anti-ASNS	Santa Cruz	Cat#sc-376151, RRID: AB_11012145
rabbit anti-GLUL	Santa Cruz	Cat#sc-74430, RRID: AB_1127501
rabbit anti-SOD2	Cell Signaling	Cat#13141S, RRID: AB_2636921
mouse anti-SOD2	Thermo Fisher Scientific	Cat#MA1-106, RRID: AB_2536812
mouse anti-RGS4	Santa Cruz	Cat#sc-398348
rabbit anti-UBR2	Abcam	Cat#ab217069
rabbit anti-UBR1	Abcam	Cat#ab138267
mouse anti-UBR1	Santa Cruz	Cat#sc-515753
rabbit anti-GSK3α	Cell Signaling	Cat#4818, RRID: AB_10831511
mouse anti-ATP5A	Santa Cruz	Cat#sc-136178, RRID: AB_2061764
rabbit anti-P62/SQSTM1	Cell Signaling	Cat#5114, RRID: AB_10624872
rabbit anti-SOD2, Lys68-specific	Abcam	Cat#ab137037, RRID: AB_2784527
rabbit anti-V5-tag	Cell Signaling	Cat#13202, RRID: AB_2687461
rabbit anti-p70S6K	Cell Signaling	Cat#9203, RRID: AB_331676
rabbit anti-p-p70S6K	Cell Signaling	Cat#9205, RRID: AB_330944
mouse anti-PSMA4	Santa Cruz	Cat#sc-271297, RRID: AB_10608330
Anti-mouse IgG HRP	Santa Cruz	Cat#sc-516102, RRID: AB_2687626
Anti-rabbit IgG HRP	Santa Cruz	Cat#sc-2357, RRID: AB_628497
Anti-mouse Alexa Fluor 488	Thermo Fisher Scientific	Cat# A-11001, RRID: AB_2534069
Anti-mouse Alexa Fluor 555	Thermo Fisher Scientific	Cat# A-21422, RRID: AB_2535844
Anti-rabbit Alexa Fluor 488	Thermo Fisher Scientific	Cat# A-11008, RRID: AB_143165
Anti-rabbit Alexa Fluor 555	Thermo Fisher Scientific	Cat# A-21428, RRID: AB_2535849
Anti-rabbit Alexa Fluor 647	Thermo Fisher Scientific	Cat #A-21245, RRID: AB_2535813
Biological samples		
PDX #3296	This paper	N/A
PDX #5639	This paper	N/A
PDX #4138	This paper	N/A
PDX #3418	This paper	N/A
PDX #5899	This paper	N/A
PDX #5910	This paper	N/A
PDX #1385	This paper	N/A
PDX #5027	This paper	N/A
PDX #4119	This paper	N/A
Chemicals, peptides, and recombinant proteins		
Asparaginase (pegaspargase)	Shire Pharmaceuticals	Oncaspar
Trypan blue	Thermo Fisher Scientific	Cat#T10282
Human FLT3	R&D systems	Cat#398-FK
Human Interleukin-7	R&D systems	Cat#206-IL-010
Human stem cell factor	R&D systems	Cat#255-SC
Human R-Spondin 3	R&D systems	Cat#3500-RS
Human Wnt3a	R&D systems	Cat#5036-WN
Puromycin	InvivoGen	Cat#ant-pr-1
Neomycin (G418/Geneticin)	InvivoGen	Cat #ant-gn-1
Blasticidin	InvivoGen	Cat#ant-bl-05
Polybrene	Merck Millipore	Cat#TR-1003-G
Lipofectamine 2000	Thermo Fisher Scientific	Cat#11668030
Dexamethasone	Sigma-Aldrich	Cat#D4902
Vincristine	Selleckchem	Cat#S1241
6-Mercaptopurine	Abcam	Cat#ab142389
Doxorubicin	Sigma-Aldrich	Cat#D1515
RIPA buffer	Merck Millipore	Cat#20-188
cOmplete Protease Inhibitor	Roche	Cat#05892970001
PhosSTOP Phosphatase Inhibitor	Roche	Cat#4906845001
Laemmli sample buffer	Bio-Rad	Cat#161-0737
b-mercaptoethanol	Sigma-Aldrich	Cat#M6250
4–20% Mini-PROTEAN^®^ TGX^™^ Precast Protein Gels	Bio-Rad	Cat#4568093
PVDF membrane	Carl Roth	Cat# T830.1
Nitrocellulose membrane, 0.45 μM	Bio-Rad	Cat#162-0115
BSA	Carl Roth	Cat#8076.4
Sure Block	LuBio Science	Cat#SB232010
GelCode Blue Safe Protein Stain	Thermo Fisher Scientific	Cat #24594
A/G agarose beads	Thermo Fisher Scientific	Cat#20423
L-Isoleucine	Sigma-Aldrich	Cat#I2752
L-Methionine	Sigma-Aldrich	Cat#M9625
L-Histidine	Sigma-Aldrich	Cat#H8000
L-Phenylalanine	Sigma-Aldrich	Cat#P2126
L-Threonine	Sigma-Aldrich	Cat#T8625
L-Tryptophan	Sigma-Aldrich	Cat#T0254
L-Lysine hydrochloride	Sigma-Aldrich	Cat#L5626
L-Valine	Sigma-Aldrich	Cat#V0500
L-Leucine	Sigma-Aldrich	Cat#L8000
Glycine	Sigma-Aldrich	Cat#G7126
L-Arginine	Sigma-Aldrich	Cat#A5131
L-Alanine	Sigma-Aldrich	Cat#A7469
L-Asparagine	Sigma-Aldrich	Cat#A0884
L-Aspartic acid	Sigma-Aldrich	Cat#A9256
L-Cystine 2HCl	Sigma-Aldrich	Cat#30200
L-Glutamic Acid	Sigma-Aldrich	Cat#G1251
L-Glutamine	Sigma-Aldrich	Cat#G3126
L-Proline	Sigma-Aldrich	Cat#P0380
L-Serine	Sigma-Aldrich	Cat#S4500
L-Hydroxyproline	Sigma-Aldrich	Cat#H5534
L-Tyrosine disodium salt dihydrate	Sigma-Aldrich	Cat#T3754
Phosphate Buffer Saline (PBS)	Thermo Fisher Scientific	Cat#14190-144
Fetal bovine serum (FBS)	Sigma-Aldrich	Cat#F7524
Human serum	Sigma-Aldrich	Cat#H4522
Leibovitz’s L-15	Thermo Fisher Scientific	Cat#11415064
Alpha-MEM	Thermo Fisher Scientific	Cat#12571063
RPMI1640 without amino acids	US Bio	Cat#R9010
DMEM	Thermo Fisher Scientific	Cat#41966029
RPMI-1640	Thermo Fisher Scientific	Cat#11875119
OptiMEM	Thermo Fisher Scientific	Cat#31985062
Polyethyleneimine	Sigma-Aldrich	Cat#408727
AgeI-HF	NEB	Cat#R3552L
MluI-HF	NEB	Cat#R3198S
BamHI-HF	NEB	Cat#R3136S
EcoRI-HF	NEB	Cat#R3101S
Sucrose	Sigma-Aldrich	Cat#S0389
Horseradish peroxidase substrate	Thermo Fisher Scientific	Cat#34577
N-acetyl-cysteine	Sigma-Aldrich	Cat#A9165
Ebselen	Sigma-Aldrich	Cat#E3520
Nicotinamide	Sigma-Aldrich	Cat#N0636
Xanthine	Sigma-Aldrich	Cat#X0626
Xanthine oxidase	Sigma-Aldrich	Cat#X4875
MnTBAP	Sigma-Aldrich	Cat#475870
Tannic Acid	Sigma-Aldrich	Cat#403040
Bortezomib	Sigma-Aldrich	Cat# 5043140001
Thapsigargin	Sigma-Aldrich	T9033
Bafilomycin	Sigma-Aldrich	Cat# SML1661
Ammonium chloride	Sigma-Aldrich	Cat#A9434
Chloroquine	Sigma-Aldrich	Cat#C6628
Potassium Chloride	Carl Roth	Cat#HN02.2
Leupeptin	Sigma-Aldrich	Cat#L2884
Pepstatin	Sigma-Aldrich	Cat#P5318
Phenylmethylsulfonylfluoride	AppliChem	Cat#A0999.0005
EDTA	AppliChem	Cat#A4892.0500
Sulfosalicylic Acid	Sigma-Aldrich	Cat#390275
Tris-HCl	Carl Roth	Cat#9090.3
Sodium chloride	Carl Roth	Cat#HN00.1
Sodium pyrophosphate tetrabasic	Sigma-Aldrich	Cat#P8010
Sodium flouride	Carl Roth	Cat#2618.1
DTT	PanReac Applichem	Cat#A2948.0010
Tween 20	Sigma-Aldrich	Cat#P9416
SDS	Carl Roth	Cat#2326.1
RevertAid Reverse Transcriptase	Thermo Fisher Scientific	Cat#EP0441
Oligo(dT)18 Primer	Thermo Fisher Scientific	Cat#SO132
dNTP Mix	Biozym	Cat#331520
iTaq Universal SYBR Green Supermix	Bio-Rad	Cat# 1725124
Paraformaldehyde	Alfa Aesar	Cat#043368
Triton X-100	Carl Roth	Cat#3051.3
Goat Serum	Thermo Fisher Scientific	Cat#31873
ProLong^™^ Diamond Antifade Mountant with DAPI	Thermo Fisher Scientific	Cat#P36962
ProLong^™^ Gold Antifade Mountant	Thermo Fisher Scientific	Cat# P10144
DAPI	Carl Roth	Cat#6335
MitoTracker^™^ Red CMXRos	Thermo Fisher Scientific	Cat#M7512
Critical commercial assays		
RNeasy Plus Mini Kit	Qiagen	Cat#74316
MycoSpy Detection Kit	Biontex Laboratories	Cat#M020
Caspase Glo 3/7 Assay	Promega	Cat#G8090
DHE Assay Kit	Abcam	Cat#ab236206
FxCycle^™^ PI/RNase Staining Solution	Thermo Fisher Scientific	Cat# F10797
GeneJet Plasmid Maxi Kit	Thermo Fisher Scientific	Cat#K0492
NucleoZOL	Macherey-Nagel	Cat#740404.200
SuperScript^™^ VILO^™^ Master Mix	Thermo Fisher Scientific	Cat#11755050
Deposited data		
Proteomics	ProteomeXchange/MassIVE	MSV000094325
Experimental models: Cell lines		
CCRF-CEM cells	ATCC	Cat#CCL-119
Jurkat cells	ATCC	Cat#TIB-152
NALM-16 cells	DSMZ	Cat#ACC-680
OCI-AML2 cells	Alex Kentsis lab	N/A
MOLT4 cells	DSMZ	Cat#ACC-362
KOPTK1 cells	ATCC	Cat# CVCL_4965
SW480 cells	ATCC	Cat#CCL-228
HCT-15 cells	ATCC	Cat#CCL-225
CCD841 cells	ATCC	Cat#CRL-1790
HEK293T cells	ATCC	Cat#CRL-3216
Oligonucleotides		
See Table S5.	This paper	N/A
Recombinant DNA		
shLuciferase	Broad RNAi Consortium	Cat# TRCN0000072243
shGSK3α #1	Broad RNAi Consortium	Cat# TRCN0000010340
shSOD1 #2	Broad RNAi Consortium	Cat# TRCN0000018344
shSOD1 #4	Broad RNAi Consortium	Cat# TRCN0000039812
shSOD2 #3	Broad RNAi Consortium, IDT	Cat# TRCN0000005942
shSOD2 #5	Broad RNAi Consortium	Cat# TRCN0000005939
shSOD3 #3	Broad RNAi Consortium	Cat# TRCN0000049077
shATE1 #1	Broad RNAi Consortium	Cat# TRCN0000034669
shATE #4	Broad RNAi Consortium	Cat# TRCN0000034672
shUBR1 #1	Broad RNAi Consortium	Cat# TRCN0000003423
shUBR1 #4	Broad RNAi Consortium	Cat# TRCN0000003424
shUBR2 #2	Kumeetal.^102^; Villa et al.^103^	N/A
shUBR2 #3	Kume et al.^102^; Villa et al.^103^	N/A
shSIRT3 #1	Broad RNAi Consortium	Cat#TRCN0000038889
shSIRT3 #2	Broad RNAi Consortium	Cat#TRCN0000038890
UBR2 cDNA	This paper	N/A
UBR1 cDNA	This paper	N/A
SOD2 cDNA	OriGene	Cat# RC202330
SOD2 Y34F mutant	Perry et al.^41^	N/A
RING WT	This paper	N/A
RING DEAD	This paper	N/A
SOD2 WT	This paper	N/A
SOD2 ΔMTS	This paper	N/A
psPAX2	Addgene.org	Cat#12260, RRID: Addgene_12260
VSV.G	Addgene.org	Cat#14888, RRID: Addgene_14888
pLX304	Addgene.org	Cat#25890
pLX302	Addgene.org	Cat#25896
pLKO.1-puro	Addgene.org	Cat# 8453
pLKO.1-blasti	Addgene.org	Cat# 26655
FBXW7 wild-type	Addgene.org	Cat# 16652
FBXW7 R465C mutant	Addgene.org	Cat# 16653
FBXW7 wild-type	GeneCopoeia	CS-T3614-LX304-01-B
FBXW7 R465C mutant	GeneCopoeia	EX-OL03574-LX304-B
DN-PSMA4	Choietal., 2016^77^	N/A
STUB1	This paper	N/A
Software and algorithms		
Photoshop	Adobe	N/A
Illustrator	Adobe	N/A
ImageQuant	Cytiva	N/A
R Studio	R Studio	N/A
MATLAB	The MathWorks	N/A

## Highlights

- SOD2 has a previously unrecognized dismutase-independent function
- It regulates proteasomal degradation through the E3 ubiquitin ligases UBR1 and UBR2
- SOD2-driven protein breakdown promotes cancer cell fitness upon amino acid scarcity
- In resistant human leukemia, inhibition of SOD2 induces asparaginase sensitization

## RESOURCE AVAILABILITY

### Lead contact

Requests for further information and resources should be directed to and will be fulfilled by the lead contact, Laura Hinze (hinze.laura@mh-hannover.de).

### Materials availability

- All plasmids and cell lines generated by this study are available upon request to the lead contact.
- Patient-derived leukemia specimens are available to investigators who can demonstrate appropriate institutional protections for human subjects research in collaboration with University Hospital Zurich, contact person: Beat Bornhauser (beat.bornhauser@kispi.uzh.ch).

### Data and code availability

- Data for the CRISPR-Cas9 loss-of-function screen has been previously reported in the following publication: https://doi.org/10.1016/j.ccell.2019.03.004.
- Proteome data are available via ProteomeXchange/MassIVE. Database: MSV000094325.
- This paper does not report any original code.
