# Supplementary material for: SOD2 is a regulator of proteasomal degradation promoting an adaptive cellular starvation response
Source: Cell Rep. Author manuscript; Available in PMC 2025 May 21. (PMC12094083; doi:10.1016/j.celrep.2025.115434)
Supplement: 1 [file NIHMS2076693-supplement-1.pdf]

Cell Reports, Volume *44*

Supplemental information

SOD2 is a regulator of proteasomal degradation promoting an adaptive cellular starvation response

Nurul Khalida Ibrahim, Sabine Schreek, Buesra Cinar, Anna Sophie Stasche, Su Hyun Lee, Andre Zeug, Tim Dolgner, Julia Niessen, Evgeni Ponimaskin, Halyna Shcherbata, Beate Fehlhaber, Jean-Pierre Bourquin, Beat Bornhauser, Martin Stanulla, Andreas Pich, Alejandro Gutierrez, and Laura Hinze

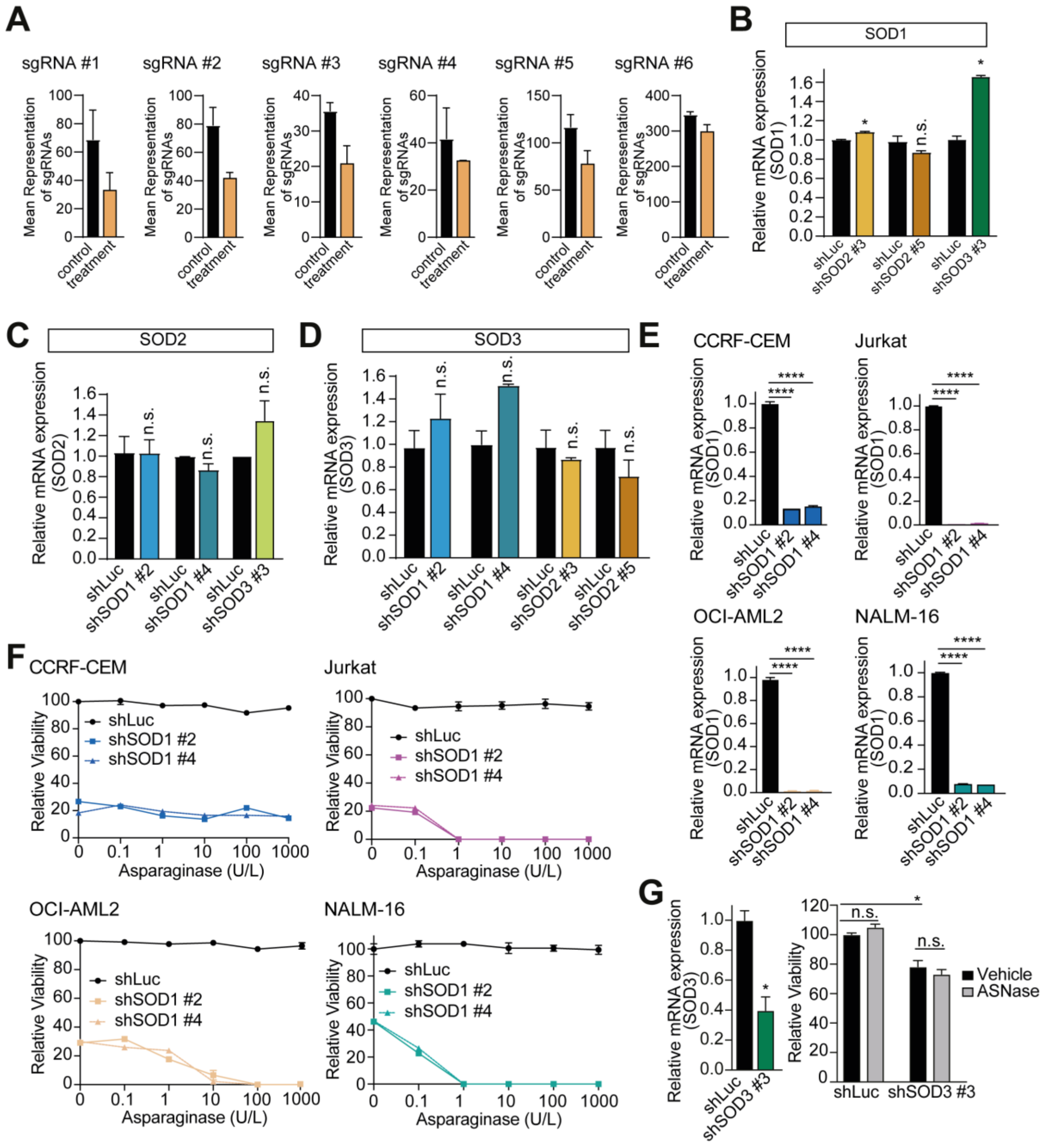

Figure S1. Isoform Specificity of SOD shRNAs, and lack of SOD1, or SOD3 inhibition on asparaginase response. Related to Figure 1 and Figure 2.

(A) Mean representation of SOD2 sgRNAs in control and treated condition of the genome-wide CRISPR screen shown in Table S1.

(B-D) Cells were transduced with indicated shRNAs. The effect of a SOD2 or SOD3 knockdown on SOD1 (B), SOD1 or SOD3 knockdown on SOD2 (C), SOD1, and SOD2 knockdown on SOD3 (D) was evaluated by RT-qPCR in biological duplicates.

(E) CCRF-CEM, Jurkat, OCI-AML2, and NALM-16 cells were transduced with indicated shRNAs, and knockdown efficiency was assessed by RT-qPCR analysis in biological duplicates.

(F) Cells from (E) were treated with the indicated doses of asparaginase, and relative viability was assessed after 8 days of treatment in biological triplicates. Cell counts were normalized to shLuc-transduced, vehicle-treated cells.

(G) Jurkat cells were transduced with shSOD3, and knockdown efficiency was assessed with RT-qPCR analysis in biological duplicates (left). Cells were then treated with 100 U/L of asparaginase, and relative viability was assessed after 6 days in biological duplicates (right). Cell counts were normalized to shLuc-transduced, vehicle-treated cells.

All error bars represent SEM. **** $p \leq 0.0001$; * $p < 0.05$; n.s. $p \geq 0.05$ by two-sided Student's t-test with Welch adjustment (B-D, G left) and one-way ANOVA with Dunnett's adjustment for multiple comparisons (E and G right). See also Table S1.

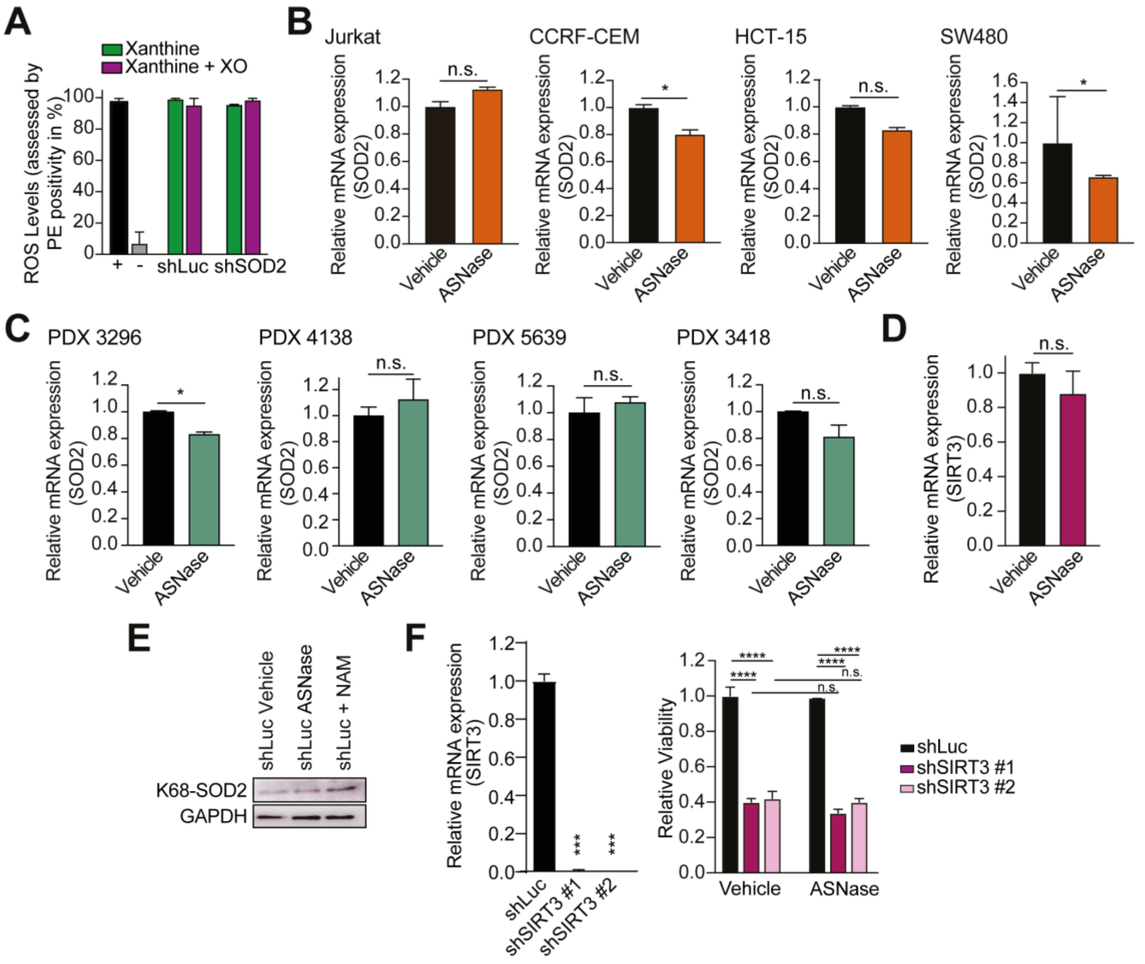

Figure S2. Levels of SOD2 in the presence of asparaginase treatment in different cancer cell lines or patient-derived xenografts (PDXs), and the role of SOD2 in the SIRT3 pathway. Related to Figure 3.

(A) Cells were transduced with the indicated constructs, and ROS levels were determined by using the DHE assay kit upon treatment with 1 mM xanthine or 1 mM xanthine plus 0.01 U/mL xanthine oxidase (XO) in biological duplicates.

(B-C) Indicated cells were treated with vehicle or asparaginase (100 U/L) for 48 hours in biological duplicates, and mRNA expression of SOD2 was assessed by RT-qPCR.

(D) Jurkat cells were treated with vehicle or asparaginase (100 U/L) in biological duplicates. After 48 hours of treatment, mRNA levels of SIRT3 were assessed by RT-qPCR.

(E) Jurkat cells were transduced with shLuc and treated with vehicle, 100 U/L asparaginase, or nicotinamide (10 mM) for 48 hours. Levels of K68-SOD2 and GAPDH were assessed by Western blot analysis.

(F) Jurkat cells were transduced with indicated shRNAs, and mRNA levels were assessed by RT-qPCR in biological duplicates (left). Viability was assessed after 4 days of treatment with vehicle or 100 U/L of asparaginase in biological duplicates (right). Cell counts were normalized to shLuc-transduced, vehicle-treated cells.

All error bars represent SEM. **** $p \leq 0.0001$; *** $p \leq 0.001$; * $p < 0.05$; n.s. $p \geq 0.05$ by two-sided Student’s t-test with Welch adjustment (B-D) and one-way ANOVA with Dunnett’s adjustment for multiple comparisons (F).

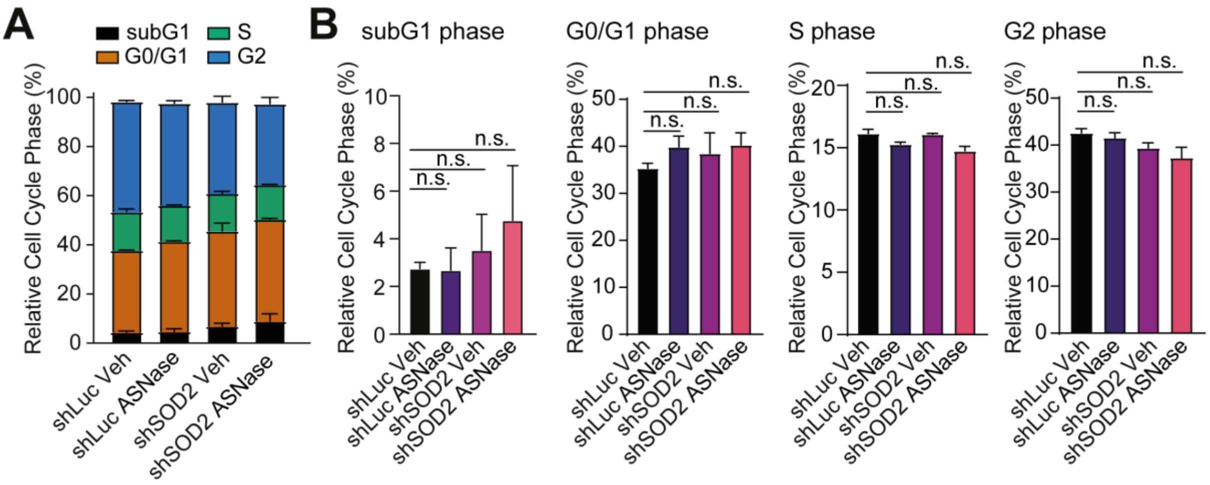

Figure S3. The effect of SOD2 inhibition and starvation on the cell cycle. Related to Figure 3.

(A) Cells were transduced with indicated shRNAs and treated with vehicle or 100 U/L asparaginase. Cell cycle analysis was conducted after 48 hours of treatment by flow cytometry in biological duplicates.

(B) Statistical analysis of the cell cycle analysis from (A) for each cell cycle phase.

All error bars represent SEM. n.s. $p \geq 0.05$ by a one-way ANOVA with Dunnett's adjustment for multiple comparisons (B).

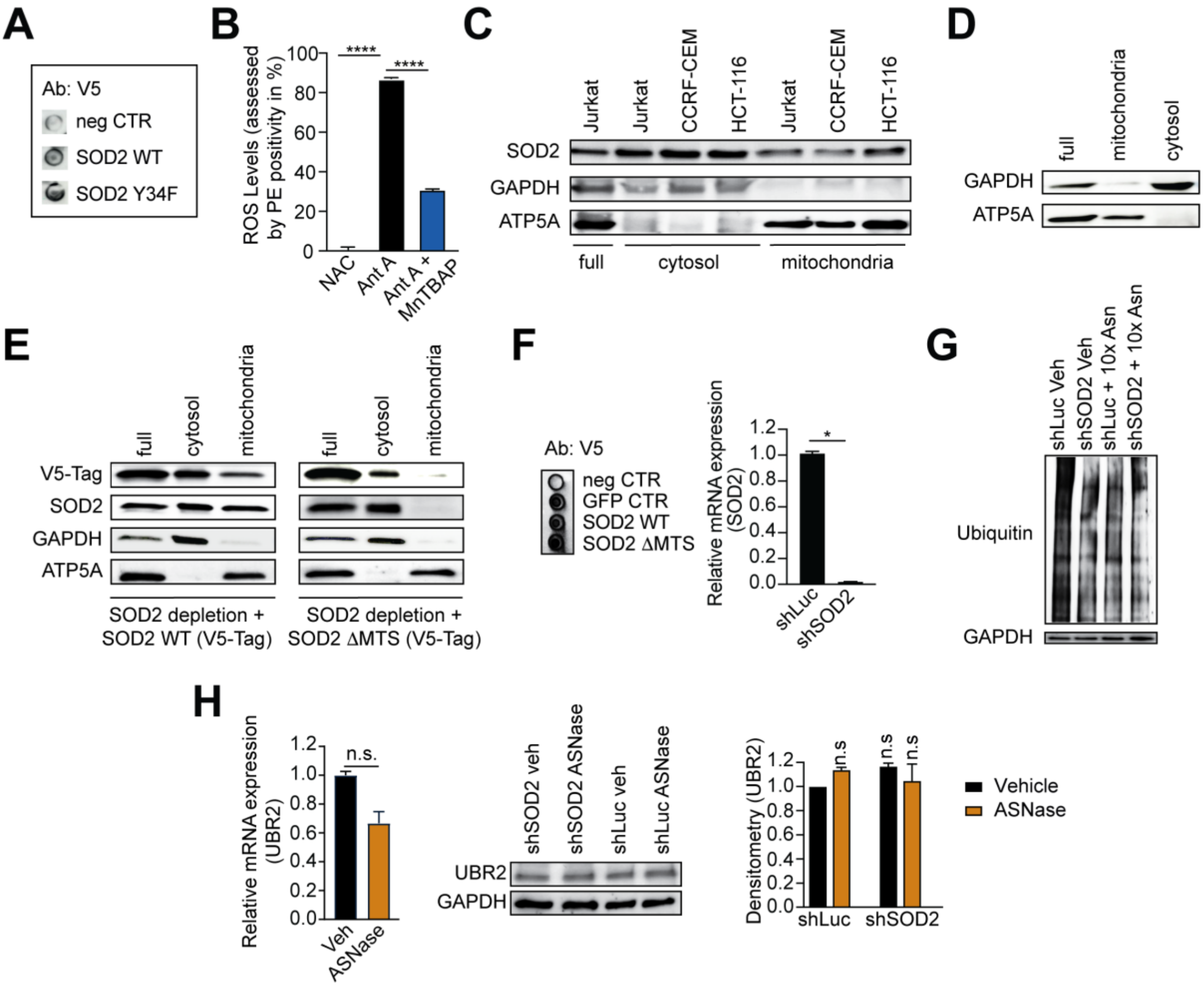

Figure S4. ROS assays, subcellular fractionations, and levels of UBR2. Related to Figure 4.

(A) Cells from the experiment shown in Figure 4A. Dot blot shows negativity for a V5 stain in untransduced Jurkat control cells, but positivity in SOD2 WT and SOD2 Y34F transduced cells confirms the expression of the respective constructs.

(B) Cells were treated as indicated, and ROS levels were determined using the DHE assay in biological duplicates. N-acetyl-cysteine served as the negative control. Antimycin A was used as a positive control, triggering extensive ROS production.

(C-D) Indicated cell lines were separated in cytosolic and mitochondrial fractions, and the indicated protein levels were detected. Note that GAPDH is a protein abundant in the cytosol and ATP5A in the mitochondria, thus demonstrating the efficacy of the fractionation.

(E) Upon SOD2 depletion, Jurkat cells were transduced with SOD2 WT or ΔMTS-SOD2 construct, lacking the mitochondrial targeting sequence (MTS) of SOD2. Cells were then separated into cytosolic and mitochondrial fractions, and the indicated protein levels were detected. Note that SOD2 WT and ΔMTS-SOD2 constructs express a V5-Tag.

(F) Dot blot shows negativity for a V5 stain in Jurkat control cells, and positivity in shSOD2 #5 cells transduced with pLX304 GFP (positive control), SOD2 WT construct, or ΔMTS-SOD2 construct, confirming the expression of the respective constructs (left). The effect of SOD2 knockdown shown in Figure S4E-F was evaluated by RT-qPCR in biological duplicates (right).

(G) Cells were transduced with shLuc or shSOD2 and treated with vehicle or with 10x asparagine for 24 hours.

(H) Jurkat cells were treated with 100 U/L asparaginase for 48 hours, and UBR2 mRNA levels were measured by RT-qPCR in biological duplicates (left). Jurkat cells were transduced with indicated shRNAs and treated with vehicle or 100 U/L asparaginase for 48 hours. Levels of UBR2 and GAPDH were assessed by Western Blot analysis (middle). Densitometry analysis was performed for the indicated western blot in biological duplicates by quantifying the target band (UBR2) and then normalizing to its respective GAPDH (right).

All error bars represent SEM. **** $p \leq 0.0001$, * $p < 0.05$; n.s. $p \geq 0.05$ by two-sided Student's t-test with Welch adjustment (F, H left) and one-way ANOVA with Dunnett's adjustment for multiple comparisons (B, H right).

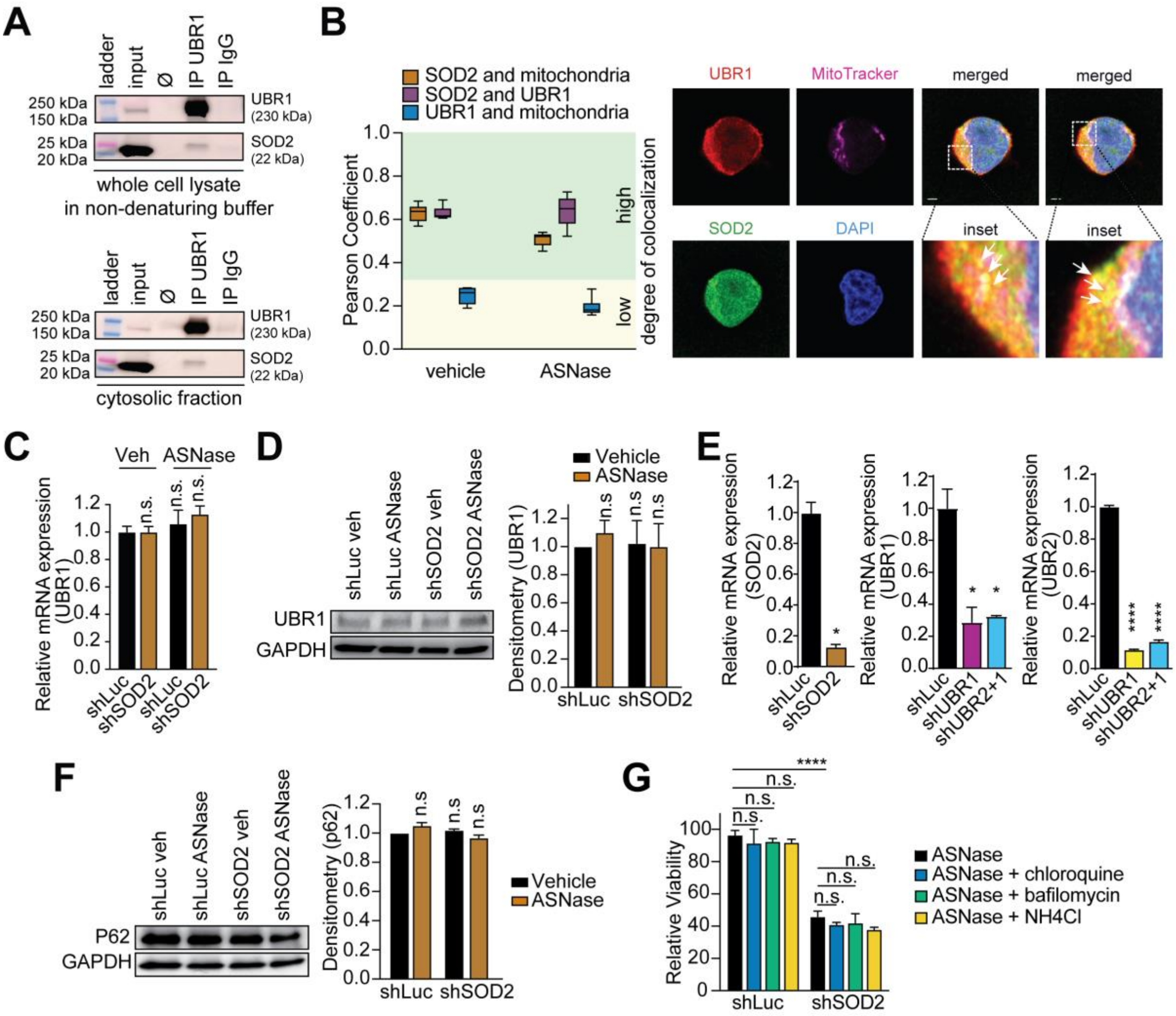

Figure S5. Interaction of SOD2 with UBR1, and the role of autophagy in the SOD2-mediated starvation response. Related to Figures 5-7.

(A) Co-immunoprecipitation of UBR1 with SOD2 in Jurkat cells in whole cell lysate (top panel) and cytosolic cell fraction (bottom panel). Ø represents an empty lane to avoid artificial spillover from the input or IP lane.

(B) Jurkat cells were treated with vehicle or 100 U/L asparaginase for 48 hours (Left). The co-localization of indicated targets was assessed by the Pearson Correlation Coefficient and ranked based on their degree of colocalization. Representative images using super-resolution microscopy (Right). Arrows indicate the colocalization of UBR1 and SOD2. Scale bar: 2 μm.

(C) Jurkat cells were transduced with the indicated shRNAs, treated with vehicle or asparaginase (100 U/L) for 48 hours, and the expression of UBR1 was assessed by RT-qPCR analysis in biological duplicates.

(D) Jurkat cells were transduced with the indicated shRNAs, treated with vehicle or asparaginase (100 U/L) for 48 hours after a confirmed knockdown, and the protein levels of UBR1 and GAPDH were assessed by Western blot. Densitometry analysis was performed in biological duplicates for UBR1 levels and normalized to their respective GAPDH signal.

(E) Jurkat cells were transduced with the indicated shRNAs, and the expression of the indicated targets was assessed by RT-qPCR analysis in biological duplicates.

(F) Jurkat cells were transduced with the indicated shRNAs, treated with vehicle or asparaginase (100 U/L) for 48 hours after a confirmed knockdown, and the protein levels of P62 and GAPDH were assessed by Western blot. Densitometry analysis was performed in biological duplicates for P62 levels and normalized to their respective GAPDH signal.

(G) Jurkat cells were transduced with the indicated shRNAs, treated with asparaginase with and without chloroquine (10 µM), bafilomycin (50 nmol/L), and NH_4Cl (1 mM) for 48 hours in biological triplicates, and relative viability was assessed by counting viable cells. Cell counts were normalized to shLuc-transduced, vehicle-treated cells.

All error bars represent SEM. **** $p \leq 0.0001$; *$p < 0.05$; n.s. $p \geq 0.05$ by a one-way ANOVA with Dunnett's adjustment for multiple comparisons (C-G) and two-sided Student's t-test with Welch adjustment (E left panel).

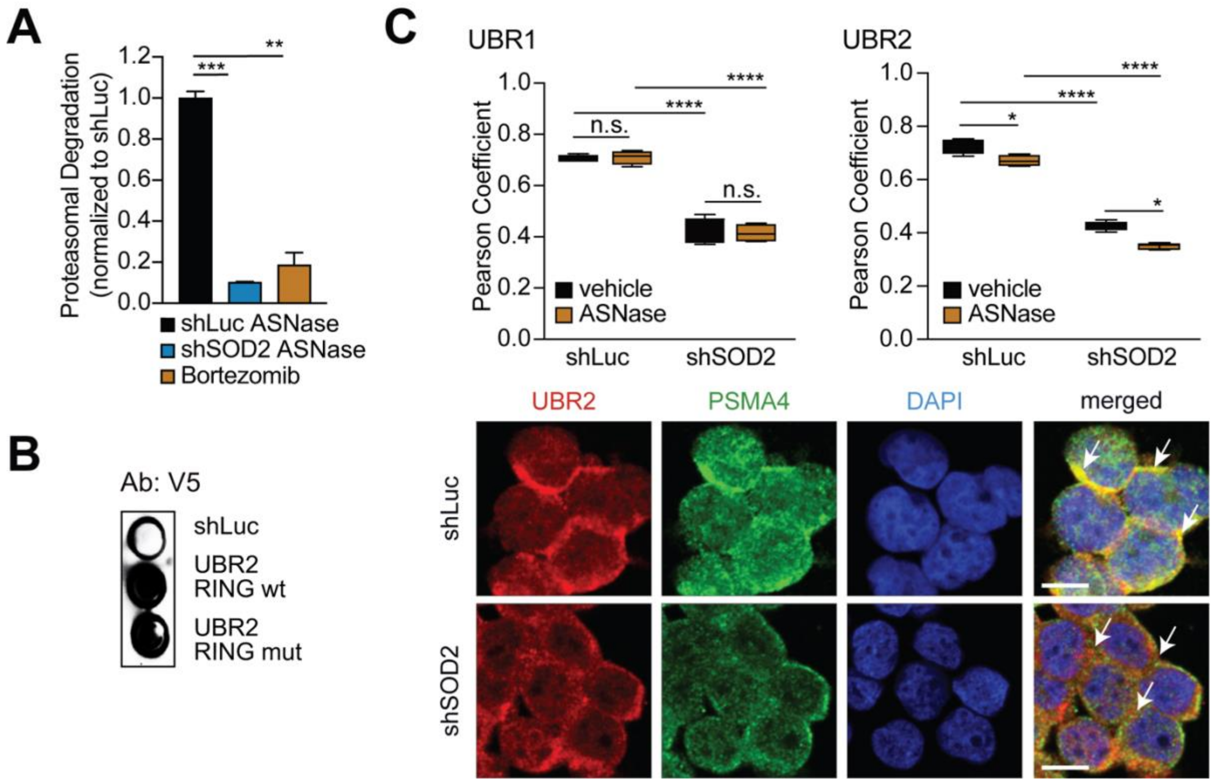

Figure S6. Loss of SOD2 inhibits colocalization of UBRs with the proteasome. Related to Figure 7.

(A) Jurkat cells were transduced with shLuc, or shSOD2 #5, followed by treatment with 1000 U/L asparaginase to induce starvation. Jurkat cells treated with bortezomib (20 nM for 5 hours) served as a positive control for sufficient inhibition of proteasomal degradation.

(B) Jurkat cells were transduced with the indicated constructs, and treated with asparaginase (1000 U/L) for 24 hours. Dot blot shows negativity for a V5 stain in shLuc cells control cells, and positivity in cells transduced with pLX304 UBR2 RING wildtype (wt), and UBR2 RING mutant (mut), confirming the expression of the respective constructs.

(C) Jurkat cells were transduced as indicated, and treated with vehicle or 100 U/L asparaginase for 48 hours. Immunofluorescence staining was observed using a Zeiss LSM780 microscope. The colocalization of indicated targets was assessed by the Pearson Correlation Coefficient. Representative images of vehicle-treated cells with arrows indicating colocalization of UBR2 and SOD2 in the presence of the shLuc non-targeting control, or the inhibition of colocalization upon knockdown of SOD2. Scale bar: 10 μm.

All error bars represent SEM. **** $p \leq 0.0001$; *** $p \leq 0.001$; ** $p \leq 0.01$; * $p < 0.05$; n.s. $p \geq 0.05$ by a one-way ANOVA with Dunnett's adjustment for multiple comparisons (A, C).

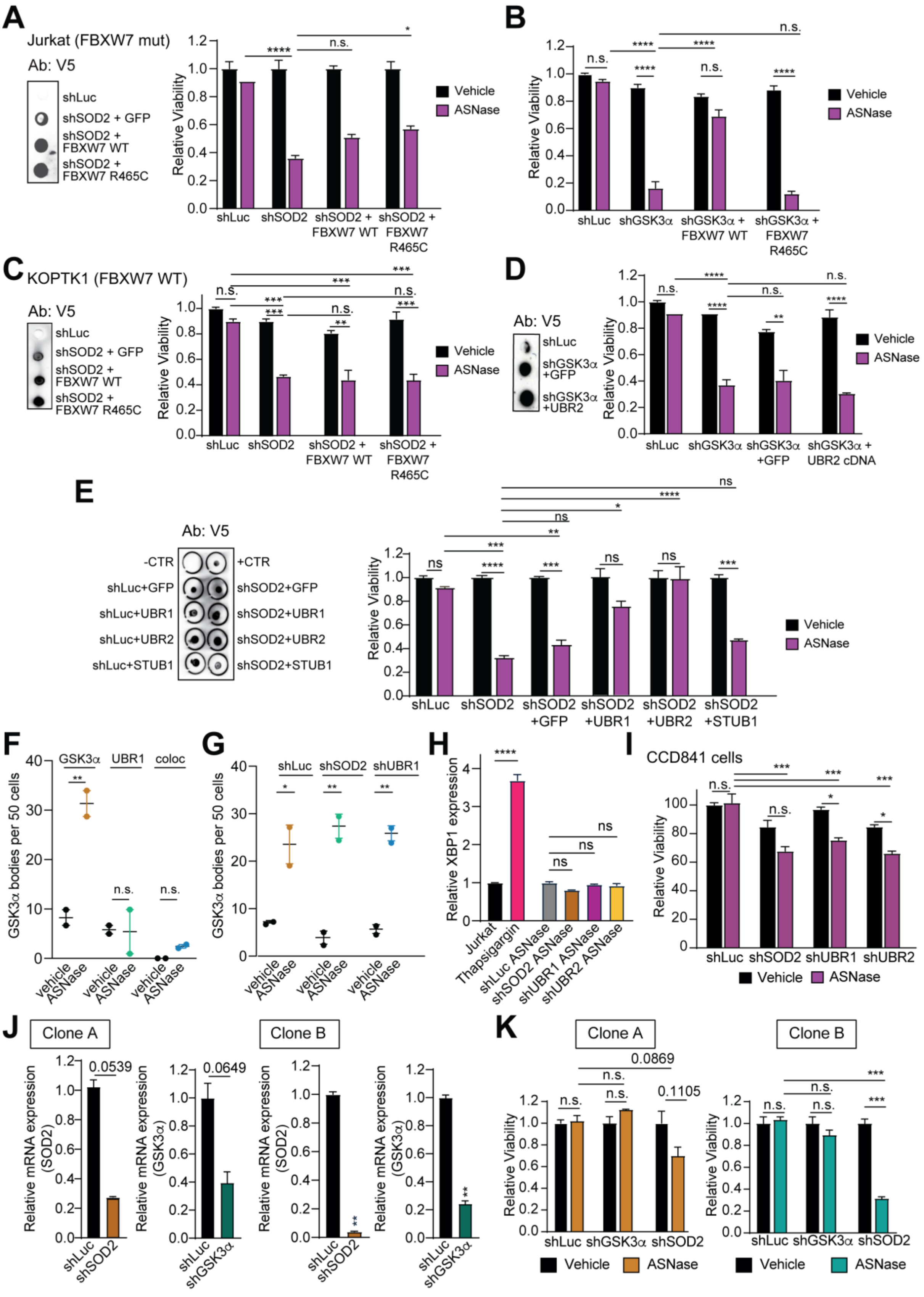

Figure S7. Association of SOD2-mediated protein degradation with WNT/STOP and UPRer. Related to Figure 7.

(A) Jurkat cells were transduced with shLuc or shSOD2 in addition to an FBXW7 wild-type (WT) or FBXW7 R465C mutant construct. After 8 days of treatment with vehicle or asparaginase (100 U/L) in biological duplicates, relative viability was assessed by counting viable cells. Cell counts were normalized to shLuc-transduced, vehicle-treated cells. Dot blot shows negativity for a V5 stain in shLuc control cells, and positivity in shSOD2 cells transduced with pLX304-GFP, pLX304-FBXW7 WT, or pLX304-R465C, confirming the expression of the respective constructs.

(B) Jurkat cells were transduced with shLuc or shGSK3α in addition to an FBXW7 WT or FBXW7 R465C mutant construct. After 8 days of treatment with vehicle or asparaginase (100 U/L) in biological duplicates, relative viability was assessed by counting viable cells. Cell counts were normalized to shLuc-transduced, vehicle-treated cells.

(C) KOPTK1 cells were transduced with shLuc or shSOD2 in addition to an FBXW7 wild-type (WT) or FBXW7 R465C mutant construct. After 8 days of treatment with vehicle or asparaginase (100 U/L) in biological duplicates, relative viability was assessed by counting viable cells. Cell counts were normalized to shLuc-transduced, vehicle-treated cells. Dot blot shows negativity for a V5 stain in shLuc control cells, and positivity in shSOD2 cells transduced with pLX304-GFP, pLX304-FBXW7 WT, or pLX304-FBXW7 R465C, confirming the expression of the respective constructs.

(D) Jurkat cells were transduced with shLuc or shGSK3α in addition to a pLX304-GFP control vector or the UBR2 cDNA. Relative viability was assessed as in (C). Dot blot shows negativity for a V5 stain in shLuc control cells, and positivity in shGSK3α cells transduced with pLX304-GFP, or pLX304-UBR2, confirming the expression of the respective constructs.

(E) Jurkat cells were transduced with shLuc or shSOD2 in addition to a pLX304-GFP control vector, UBR1, UBR2, or STUB1 cDNA. Relative viability was assessed as in (C). Dot blot shows negativity for a V5 stain in shLuc control cells, and positivity in cells transduced with additional pLX304 constructs, confirming their respective expressions. Note that for ease of illustration, viability upon overexpression of the respective constructs is only displayed in shSOD2 deficient cells as the overexpression in shLuc cells did not show any significant differences.

(F) Jurkat cells were treated with vehicle or asparaginase (100 U/L) for 48 hours. Cells were fixed, stained for GSK3α and UBR1, and analyzed by confocal immunofluorescence microscopy. The number of foci positive for GSK3α, UBR1, or both markers colocalized was assessed in biological duplicates.

(G) Jurkat cells were transduced with the indicated constructs, and treated with vehicle or asparaginase (100 U/L) for 48 hours. Cells were fixed, stained for GSK3α, and analyzed by confocal immunofluorescence microscopy. The number of foci positive for GSK3α was assessed in biological duplicates.

(H) Jurkat cells were transduced with the indicated constructs and treated with 100 U/L asparaginase, or treated with 100 nM thapsigargin only as a positive control. Effects on mRNA expression levels for spliced XPB1 were assessed by RT-qPCR in biological duplicates.

(I) CCD841 cells were transduced with indicated shRNAs, and treated with vehicle or asparaginase (100 U/L) for 48 hours in biological duplicates. Viability was assessed by counting viable cells, and cell counts were normalized to shLuc-transduced, vehicle-treated cells.

(J) Clones were transduced with indicated shRNAs, and knockdown efficiency was assessed by RT-qPCR.

(K) Cells from (J) were treated with asparaginase (100 U/L) for 48 hours in biological duplicates. Viability was assessed by counting viable cells, and cell counts were normalized to shLuc-transduced, vehicle-treated cells.

All error bars represent SEM. **** $p \leq 0.0001$; *** $p \leq 0.001$; ** $p \leq 0.01$; * $p < 0.05$; n.s. $p \geq 0.05$ by two-sided Student's t-test with Welch adjustment (J) and one-way ANOVA with Dunnett's adjustment for multiple comparisons (A-I, K).

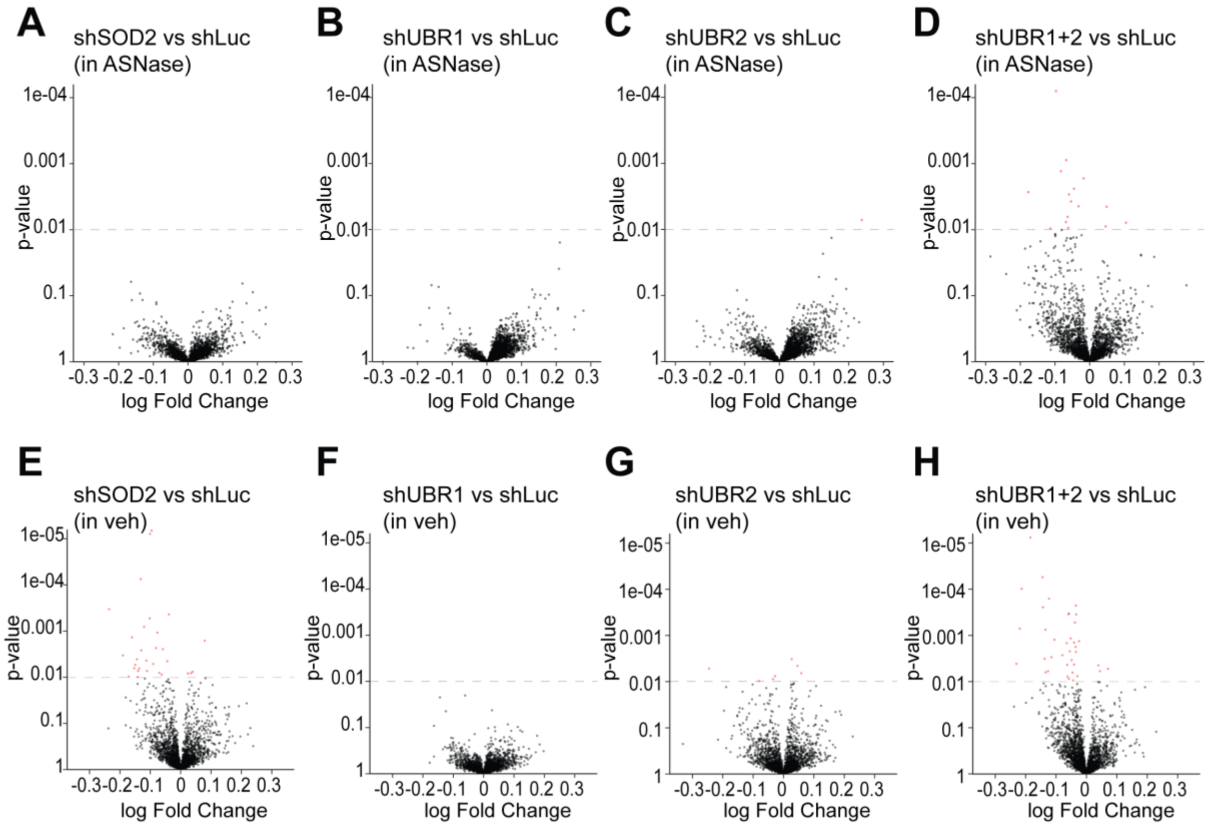

Figure S8. Inhibition of SOD2-mediated protein degradation does not have consistent effects on individual proteins. Related to Figure 7.

(A-H) Jurkat cells were transduced with the indicated constructs in biological triplicates. Upon validation of an efficient knockdown, cells were treated with vehicle (PBS) or asparaginase (100 U/L) for 48 hours, followed by protein extraction and subsequent mass spectrometry proteomics. Differential expression analysis was performed for the indicated comparisons in vehicle or asparaginase-treated conditions. For statistical analysis, limma functions, voom, and eBayes (trend=TRUE, robust=TRUE) were used to calculate and retrieve log-fold change and p-value. Proteome data are available via ProteomeXchange/MassIVE with the identifier MSV000094325.

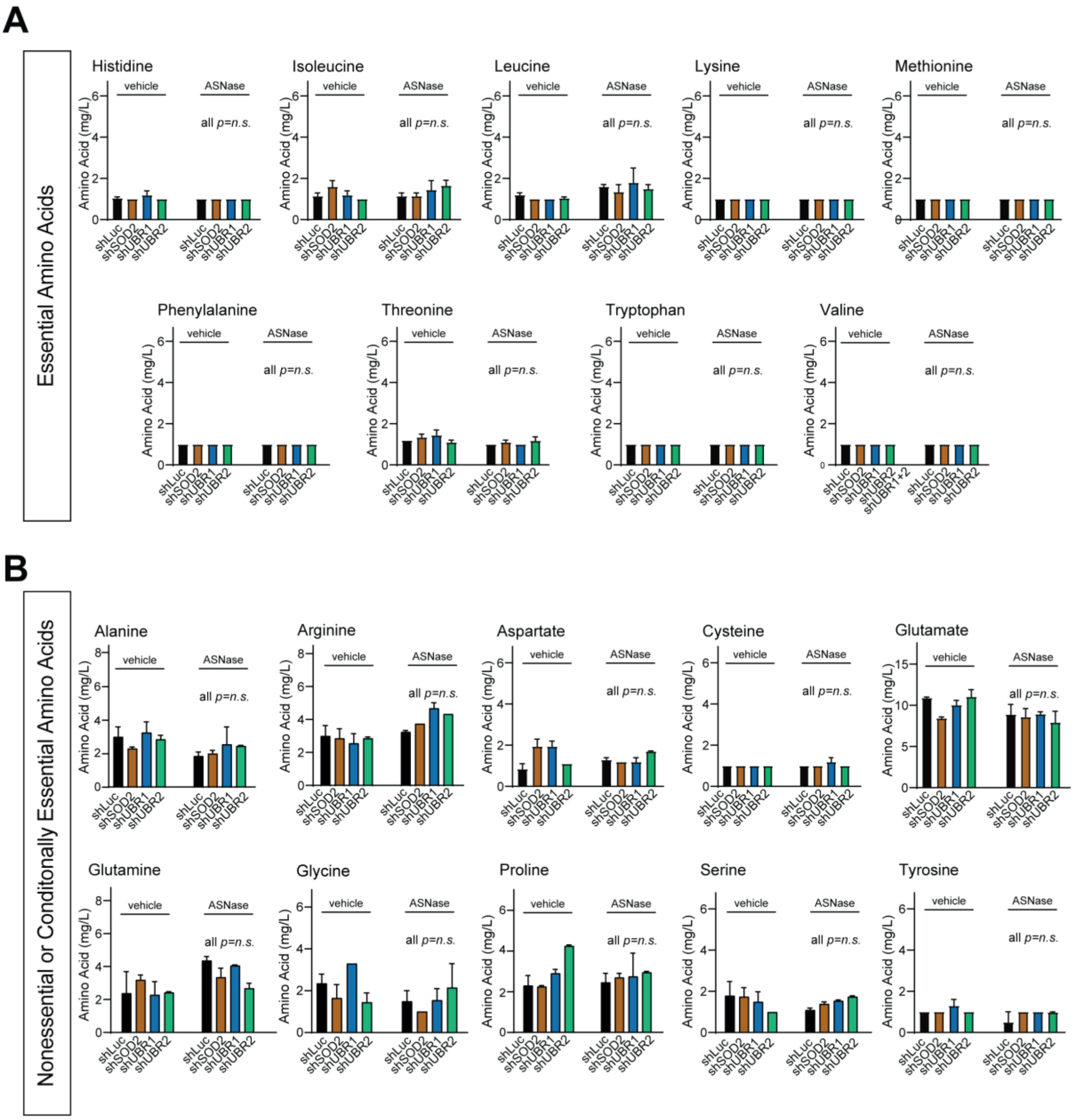

Figure S9. Asparaginase treatment does not impact other levels of amino acids in cells with SOD2-, UBR1, or UBR-2 knockdown. Related to Figure 7.

(A-B) Jurkat cells were treated with vehicle or asparaginase (10 U/L) for 48 hours. Levels of essential (A), nonessential, or conditionally essential (B) amino acids in cells were quantified by LC/MS-MS in biological triplicates.

All error bars represent SEM. n.s. $p \geq 0.05$ by one-way ANOVA with Dunnett's adjustment for multiple comparisons (A-B).

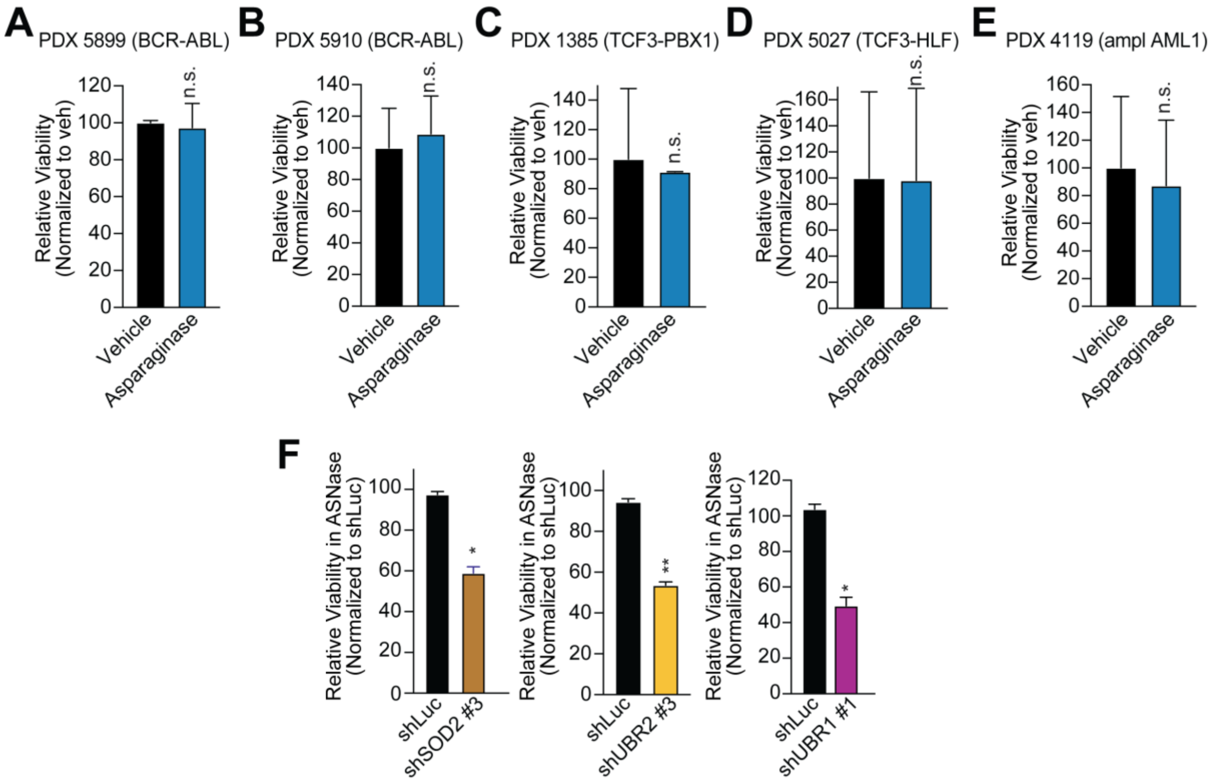

Figure S10. Asparaginase response of human PDX leukemia specimens. Related to Figure 7.

(A-E) The panel of human PDX models shown in Figure 7 and Table S3 were treated with asparaginase (100 U/L) or vehicle control for 48 hours, and viability was assessed by trypan blue vital dye exclusion. The relative viability in asparaginase of each case is shown. Cells were classified as resistant based on the results of this analysis.

(F) Jurkat cells were transduced indicated constructs, and relative viability was assessed in biological duplicates after 48 hours of treatment.

All error bars represent SEM. ** $p \leq 0.01$; *$p < 0.05$; n.s. $p \geq 0.05$ by a two-sided Student's t-test with Welch adjustment.
